# Intersectional Disparities in Emergency Medicine Residents’ Performance Assessments by Race, Ethnicity, and Sex

**DOI:** 10.1001/jamanetworkopen.2023.30847

**Published:** 2023-09-21

**Authors:** Elle Lett, Nguyen Khai Tran, Nkemjika Nweke, Mytien Nguyen, Jung G. Kim, Eric Holmboe, William McDade, Dowin Boatright

**Affiliations:** 1 Health Systems and Population Health, University of Washington School of Public Health, Seattle; 2 Center for Anti-Racism and Community Health, University of Washington School of Public Health, Seattle; 3 Perelman School of Medicine, University of Pennsylvania, Philadelphia; 4 The PRIDE Study/PRIDEnet, Stanford University School of Medicine, Palo Alto, California; 5 St George’s University School of Medicine, St George, Grenada; 6 Yale School of Medicine, New Haven, Connecticut; 7 Department of Health System Science, Kaiser Permanente Bernard J. Tyson School of Medicine, Pasadena, California; 8 Accreditation Council for Graduate Medical Education, Chicago, Illinois; 9 Ronald O. Perelman Department of Emergency Medicine, New York University, New York

## Abstract

**Importance:**

Previous studies have demonstrated sex-specific disparities in performance assessments among emergency medicine (EM) residents. However, less work has focused on intersectional disparities by ethnoracial identity and sex in resident performance assessments.

**Objective:**

To estimate intersectional sex-specific ethnoracial disparities in standardized EM resident assessments.

**Design, Setting, and Participants:**

This retrospective cohort study used data from the Association of American Medical Colleges and the Accreditation Council for Graduate Medical Education Milestones (Milestones) assessments to evaluate ratings for EM residents at 128 EM training programs in the US. Statistical analyses were conducted in June 2020 to January 2023.

**Exposure:**

Training and assessment environments in EM residency programs across comparison groups defined by ethnoracial identity (Asian, White, or groups underrepresented in medicine [URM], ie, African American/Black, American Indian/Alaska Native, Hispanic/Latine, and Native Hawaiian/Other Pacific Islander) and sex (female/male).

**Main Outcomes and Measures:**

Mean Milestone scores (scale, 0-9) across 6 core competency domains: interpersonal and communications skills, medical knowledge, patient care, practice-based learning and improvement, professionalism, and system-based practice. Overall assessment scores were calculated as the mean of the 6 competency scores.

**Results:**

The study sample comprised 128 ACGME-accredited programs and 16 634 assessments for 2708 EM residents of which 1913 (70.6%) were in 3-year and 795 (29.4%) in 4-year programs. Most of the residents were White (n = 2012; 74.3%), followed by Asian (n = 477; 17.6%), Hispanic or Latine (n = 213; 7.9%), African American or Black (n = 160; 5.9%), American Indian or Alaska Native (n = 24; 0.9%), and Native Hawaiian or Other Pacific Islander (n = 4; 0.1%). Approximately 14.3% (n = 386) and 34.6% (n = 936) were of URM groups and female, respectively. Compared with White male residents, URM female residents in 3-year programs were rated increasingly lower in the medical knowledge (URM female score, −0.47; 95% CI, −0.77 to −0.17), patient care (−0.18; 95% CI, −0.35 to −0.01), and practice-based learning and improvement (−0.37; 95% CI, −0.65 to −0.09) domains by postgraduate year 3 year-end assessment; URM female residents in 4-year programs were also rated lower in all 6 competencies over the assessment period.

**Conclusions and Relevance:**

This retrospective cohort study found that URM female residents were consistently rated lower than White male residents on Milestone assessments, findings that may reflect intersectional discrimination in physician competency evaluation. Eliminating sex-specific ethnoracial disparities in resident assessments may contribute to equitable health care by removing barriers to retention and promotion of underrepresented and minoritized trainees and facilitating diversity and representation among the emergency physician workforce.

## Key Points

**Question:**

Are their disparities by ethnoracial identity and sex in Accreditation Council for Graduate Medical Education Milestones ratings for emergency medicine residents?

**Findings:**

This retrospective cohort analysis of 16 634 assessments of 2708 emergency medicine residents in 128 training programs found evidence of sex-specific ethnoracial disparities in ratings on the Milestones assessments. These disparities increased over time across multiple Milestones assessments and were most severe for female residents of ethnoracial groups that are underrepresented in medicine (URM).

**Meaning:**

These findings suggest that intersectional disparities in Milestones assessment ratings of emergency medicine residents may be an intervenable barrier to equitable representation, particularly for female residents of ethnoracial groups that are URM, specifically in emergency medicine.

## Teaser

This retrospective cohort study evaluates Milestone assessments among emergency medicine residents in 128 US programs to identify sex-specific ethnoracial disparities.

## Introduction

Health equity is a priority for many national governing health care bodies including the US Centers for Disease Control and Prevention,^1^ the American Medical Association,^2^ and the American College of Physicians.^3^ Yet achieving a diverse health care workforce that is representative of the populations served remains a critical challenge. The benefits of a representative physician workforce include improved care access for individuals from minoritized ethnoracial groups or economically disadvantaged populations,^4,5,6^ research innovation relevant to marginalized groups,^7,8,9^ and improved clinical learning environments.^10^ These benefits are substantial in the emergency department where complex sociostructural factors determine which populations and health conditions are disproportionately represented. Emergency medicine (EM) physicians provide safety-net care to patients who may be otherwise excluded or neglected by the health care system, including patients who are uninsured,^11^ experiencing homelessness,^12^ or have comorbid psychiatric illnesses with alcohol and substance use disorders.^13^ An empathic and diverse emergency physicianship representative of the demographic composition of the populations they serve is necessary to optimize care.

Disparities in performance assessments represent a manifestation of discrimination, including institutional racism, creating a barrier to advancement for minoritized trainees. In undergraduate medical education (UME), studies have demonstrated both racial and gendered disparities in Medical Student Performance Evaluations. Assessments of female medical students and medical students of ethnoracial groups that are underrepresented in medicine (URM) were less likely to include competency-based descriptions and were more likely to include descriptions of personality traits compared with assessment of male residents who were not URM.^14^ A similar study showed that evaluations for Black medical students were less likely to emphasize exceptional ability, and for female medical students, more likely to include descriptions of personality and emotional characteristics.^15^ These studies indicate that disparities in UME evaluations relegate the strengths of racially minoritized and female trainees to psychosocial and emotional traits while de-emphasizing technical skills and competencies in comparison with White and male peers. Racial disparities in UME performance evaluations extend beyond the narrative evaluations to application elements used by residency program directors to make admissions decisions,^16^ including clinical clerkship grades and standardized summative evaluations,^17^ and Alpha Omega Alpha honor society membership.^18^

Disparities in performance assessments persist into graduate medical education and have been demonstrated in standardized resident milestone assessments for internal^19,20^ and EM.^21,22^ Similar to the UME findings, these studies show that residents who are female^19,21,22^ or from a URM group^20^ are consistently rated as less skilled than their male and non-URM counterparts. However, previous studies have been limited because they addressed racial and gender disparities^23,24^ separately in assessments and in only a fraction of training programs.

Invoking intersectionality,^25^ we argue that concomitantly estimating these disparities’ simultaneous and interactive influences is critical. This study sought to extend the previous literature by performing a retrospective cohort study of sex-specific ethnoracial discrimination in assessments of EM residents using the Accreditation Council for Graduate Medical Education (ACGME) Milestones (hereafter, Milestones) data for academic year 2014 to 2015 through academic year 2017 to 2018.

## Methods

### Study Participants and Setting

This retrospective cohort study was an analysis of Milestones assessments for residents in ACGME-accredited EM training programs from academic year 2014 to 2015 through academic year 2017 to 2018. Ratings are based on standardized Milestones reported to the ACGME and linked to resident demographic data provided by the Association of American Medical Colleges (AAMC). These characteristics include self-reported ethnoracial identity, binary sex, and United States Medical Licensing Examination Step 2 Clinical Knowledge (USMLE Step 2 CK) scores.

This study followed the Strengthening the Reporting of Observational Studies in Epidemiology (STROBE) reporting guidelines for cohort studies. The study was reviewed and deemed exempt from obtaining informed consent by the Yale University institutional review because it used only deidentified data.

### Exclusion Criteria and Analytic Cohort

We merged ACGME and AAMC data and excluded 308 residents whose records were not present in both sources. The initial data set comprised 24 718 assessments of 4283 residents at 209 EM programs in the US (eTable 1 and eFigure 1 in Supplement 1). We also excluded residents whose ethnoracial data were missing, either because the AAMC does not provide information for individuals who are not US citizens or permanent residents (n = 541) or for an unknown reason (n = 56). We excluded an additional 332 residents with unavailable USMLE Step 2 CK scores. Moreover, given that our study goal was to estimate sex-specific ethnoracial disparities, which requires a racially diverse pool of trainees, we also excluded programs that did not have at least 1 Asian and 1 URM trainee during the study period. By excluding residents from the programs that lacked diversity, we sought to avoid potentially biased results at the expense of a more highly powered analysis. This final exclusion criteria reduced the number of assessments included in the study from 20 343 to 16 634, residents from 3354 to 2708, and EM programs from 188 to 128 (eFigure 1 in Supplement 1).

### ACGME Milestones Assessments

The Milestone assessments are “competency-based developmental outcomes (eg, knowledge, skills, attitudes, and performance) that can be demonstrated progressively by residents/fellows from the beginning of their education through graduation to the unsupervised practice of their specialties.”^26^ Emergency medicine residents are assessed twice during the academic year (midyear and year-end) across 23 subcompetencies grouped into 6 core ACGME competency domains: patient care, medical knowledge, systems-based practice, practice-based learning and improvement, professionalism, and interpersonal and communication skills (eTable 2 in Supplement 1). Each subcompetency was scored from 0 to 9 across 5 Milestones levels.

### Conceptual Model

We drew on the theoretical framework of intersectionality for this study. Intersectionality was institutionalized in academia by the work of legal scholar, Kimberlé Crenshaw,^27^ and sociologist, Patricia Collins,^28^ among others. It has roots that date back as early as the social movements of the early 19th century.^29,30,31^ Intersectionality posits that sociostructural systems are interactive and that individuals with multiply marginalized identities are subject to their co-occurring and synergistic processes, manifesting ubiquitously, including in clinical learning environments. We were particularly interested in 2 of these systems—racism and sexism—exposure to which are imperfectly captured^32^ by ethnoracial and binary sex data provided by AAMC. This study quantifies sex-specific ethnoracial disparities in EM residency assessments which may be, in part, a manifestation of interpersonal, institutional, or structural racism and sexism.

### Ethnoracial Groupings

We divided the EM residents into 3 ethnoracial groups: Asian, URM, and White. In the data, residents were able to self-identify with multiple ethnoracial groups. For those who selected more than 1 group, any URM identity was prioritized, defined per AAMC guidelines as African American or Black, Hispanic or Latine, American Indian or Alaska Native, and Native Hawaiian or Other Pacific Islander. If an individual selected Asian and White, they were included as Asian in our analyses. It is important to note that race and ethnicity are conceptualized as a proxy for exposure to structural, institutional, and interpersonal racism and ethnicism, rather than an essentialist biological or cultural variable that would drive differential resident performance. We used the term *minoritized* ethnoracial groups, rather than *minority* per Harper,^33^ noting that social identities and their corresponding statuses are context dependent and that individuals are minorities only within institutions structured to facilitate the overrepresentation and privilege of Whiteness. Similarly, underrepresentation in this study and in medicine broadly, is defined according to coarse racial taxonomies that do not capture within-group heterogeneity. In particular, the Asian group comprises many subpopulations that may be over- or underrepresented in medicine. We make note that although some of these groups may not be underrepresented, they are all *minoritized* in that they are harmed by manifestations of systemic racism in the US.

### Statistical Analyses

We used hierarchical linear mixed-effects models to estimate sex-specific ethnoracial disparities in Milestone scores. The primary outcomes included the mean competency scores for the 6 competencies being assessed. Our secondary outcome was the mean assessment score for each Milestone assessment in its entirety. This choice in secondary outcome equally weighted each competency (ie, providing equal weight to patient care and medical knowledge). We fit separate models for 3-year and 4-year programs. Each model included fixed-effects for ethnoracial group (Asian, URM, White), binary sex (female/male), and time (half-years of training) as well as pairwise and tertiary interactions among those variables. The interaction terms estimated separate trajectories for each ethnoracial group−sex cross-strata and quantified the differences in those trends at each time point. We had a 3-level model with assessments (level 1) nested within EM residents (level 2) that are nested within programs (level 3). The final model had random intercepts for both residents and programs and a random slope for residents with respect to time. We also adjusted for USMLE Step 2 CK score as a measure of baseline medical knowledge prior to residency, and academic year for temporal trends in resident assessments given that each program length had 2 staggered cohorts (eTables 3 and 4 in Supplement 1).

We present linear contrasts and 95% CIs for each ethnoracial group and sex cross-strata relative to White male residents at the same time point per competency. The 95% CIs that do not include zero correspond to statistically significant results for a 2-sided hypothesis test with a type I error rate of α = .05. Because this study was the first, to our knowledge, to estimate intersectional disparities in resident milestone evaluations, it was exploratory in nature; we did not adjust for multiple comparisons.^34^ The reference group was purposely selected as comparing individuals from marginalized groups with the most privileged group that is least likely to be subject to ethnoracial- or sex-based discrimination. All analyses were conducted in R, version 4.2.1 (The R Foundation for Statistical Computing) and mixed-effects models were fit using the lme4 package.^35^ Data analyses were performed between June 2020 and January 2023.

## Results

Of the 2708 EM residents in our analysis, 1913 (70.6%) were in 3-year programs and 795 (29.4%) in 4-year programs (Table). Most of EM residents in the sample were White (n = 2012; 74.3%), followed by Asian (n = 477; 17.6%), Hispanic or Latine (n = 213; 7.9%), African American or Black (n = 160; 5.9%), American Indian or Alaska Native (n = 24; 0.9%), and Native Hawaiian or Other Pacific Islander (n = 4; 0.1%). Approximately 14.3% (n = 386) and 34.6% (n = 936) of the sample were URM residents and female, respectively. The median USMLE Step 2 CK score was 243 (IQR, 232-253). We found that 60 programs (28.7% of all programs and 3.2% of programs with assessments that met our exclusion criteria) did not have Asian or URM residents in their training program (eFigure 1 in Supplement 1).

**Table.** Baseline Characteristics of Included Emergency Medicine Residents (n = 2708)

Characteristic	Residents, No. (%)
Ethnoracial identity^a^	
African American or Black	160 (5.9)
Asian	477 (17.6)
Hispanic or Latine	213 (7.9)
American Indian or Alaska Native	24 (0.9)
Native Hawaiian or Other Pacific Islander	4 (0.1)
White	2012 (74.3)
Ethnoracial group	
Asian	465 (17.2)
URM	386 (14.3)
White	1857 (68.6)
Sex	
Female	936 (34.6)
Male	1772 (65.4)
Intersectional group	
Asian, female	158 (5.8)
Asian, male	307 (11.3)
URM, female	160 (5.9)
URM, male	226 (8.3)
White, female	618 (22.8)
White, male	1239 (45.8)
Program length^b^	
3 y	1913 (70.6)
4 y	795 (29.4)
USMLE Step 2 CK score, median (IQR)	243 (232-253)

Abbreviations: URM, underrepresented in medicine; USMLE Step 2 CK, United States Medical Licensing Examination Step 2 Clinical Knowledge.

^a^ Residents were able to self-identify as more than 1 ethnoracial group.

^b^ Number of unique residents with at least 1 evaluation in a program of the corresponding duration.

### Milestone Competency Scores

**Figure 1.**
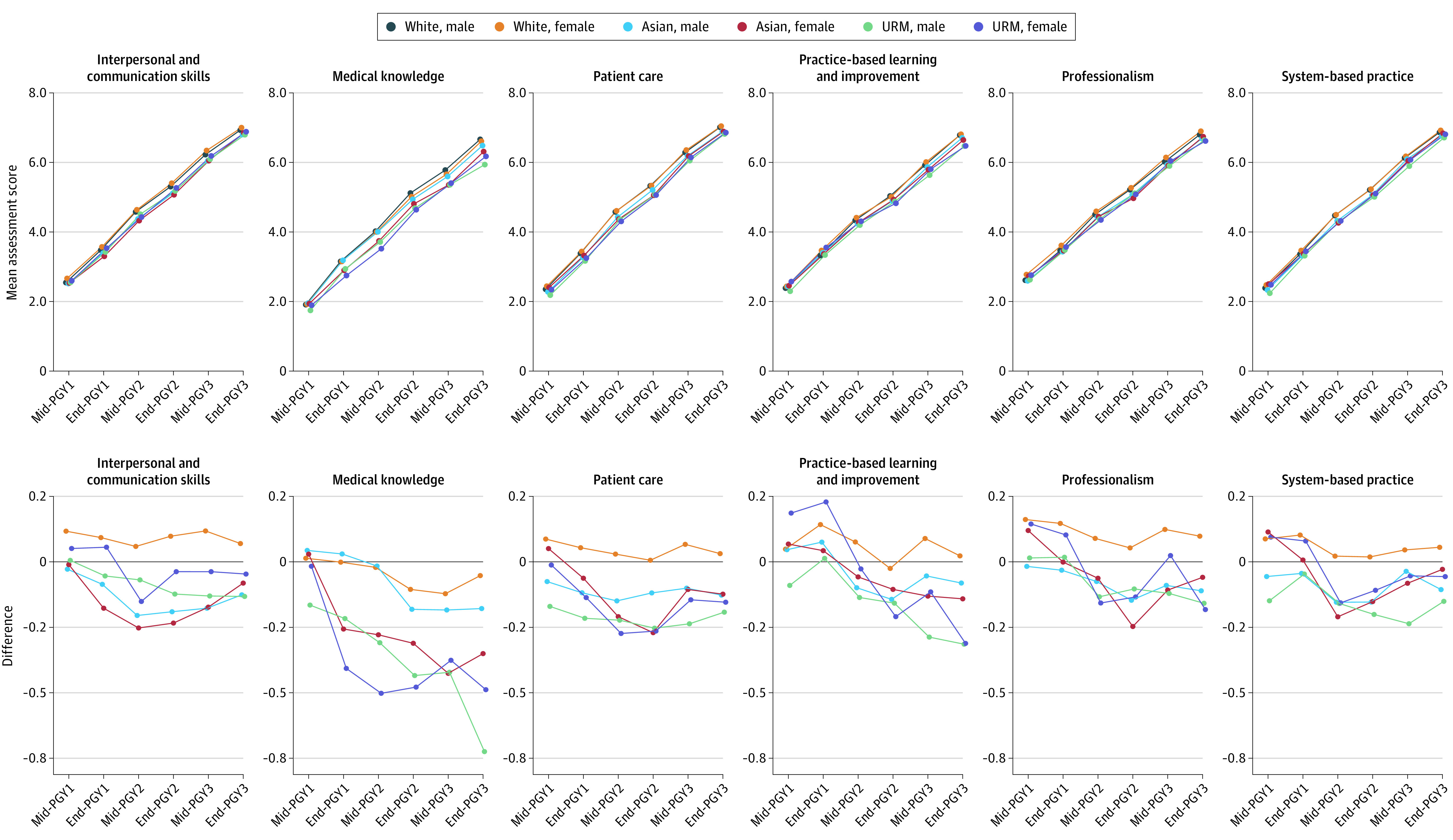
Competency Score Trajectories by Ethnoracial Group and Sex Cross-Strata, 3-Year Emergency Medicine Residency Programs Changes in the mean scores for 6 different Accreditation Council for Graduate Medical Education competencies among US emergency medicine residents in 3-y programs. Mean competency scores for each postgraduate year (PGY) time point were calculated as the mean of each subcompetency score for each corresponding competency for each racial/ethnic group and sex cross-strata. URM indicates underrepresented in medicine.

**Figure 2.**
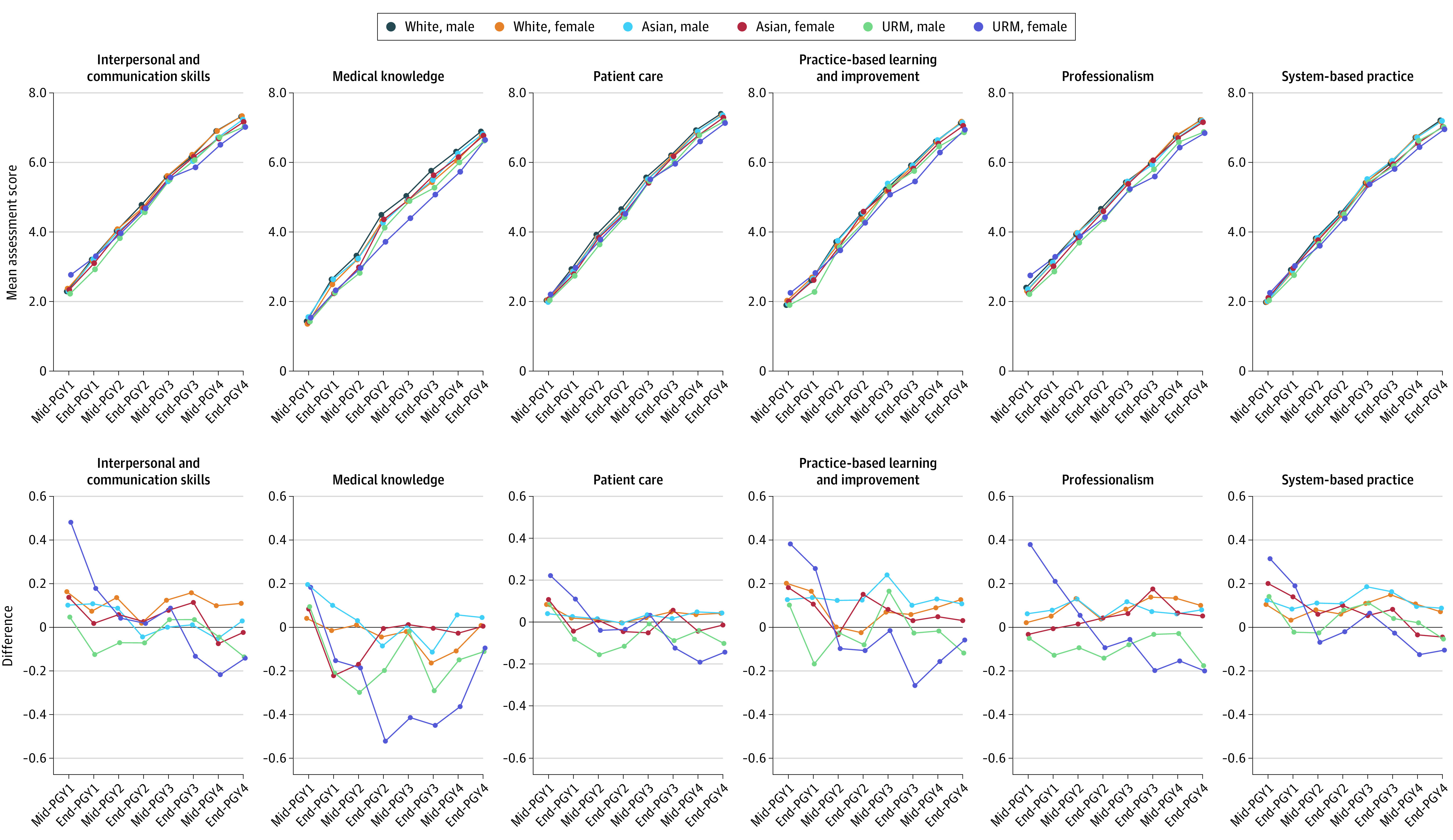
Competency Score Trajectories by Ethnoracial Group and Sex Cross-Strata, 4-Year Emergency Medicine Residency Programs Changes in the mean scores for 6 different Accreditation Council for Graduate Medical Education competencies among US emergency medicine residents in 4-y programs. Mean competency scores for each postgraduate year (PGY) time point were calculated as the mean of each subcompetency score for each corresponding competency for each racial/ethnic group and sex cross-strata. URM indicates underrepresented in medicine.

Residents in the 3-year programs were rated higher than those in 4-year programs at the mid-year assessment for postgraduate year 1 (PGY1), with similar rates of change in the mean scores over the assessment period (Figure 1; eFigure 2 in Supplement 1). However, we found that competency scores differed across ethnoracial groups, sex, and program length. In the 3-year programs, ethnoracial group−sex cross-strata had comparable ratings in all competency domains at the PGY1 midyear assessment (Figure 1; eTable 5 in Supplement 1). In the 4-year programs, URM female residents received the highest scores for all competencies except for medical knowledge in PGY1 midyear assessment (Figure 2; eTable 6 in Supplement 1). Disparities began to emerge in PGY2, with Asian and URM residents of both sexes receiving increasingly lower ratings than White male residents for all competencies at PGY2 year-end assessment. In the 4-year programs, only URM female residents received increasingly lower scores than White male residents in all competencies. The largest differences were observed in medical knowledge, particularly between URM male residents (mean [SD], 5.94 [1.45]) and White male residents (mean [SD], 6.66 [1.06]) in 3-year programs at PGY3 end-of-year, and between URM female residents (mean [SD], 3.72 [1.03]) and White male residents (mean [SD], 4.49 [1.10]) in 4-year programs at PGY2 year-end assessments.

**Figure 3.**
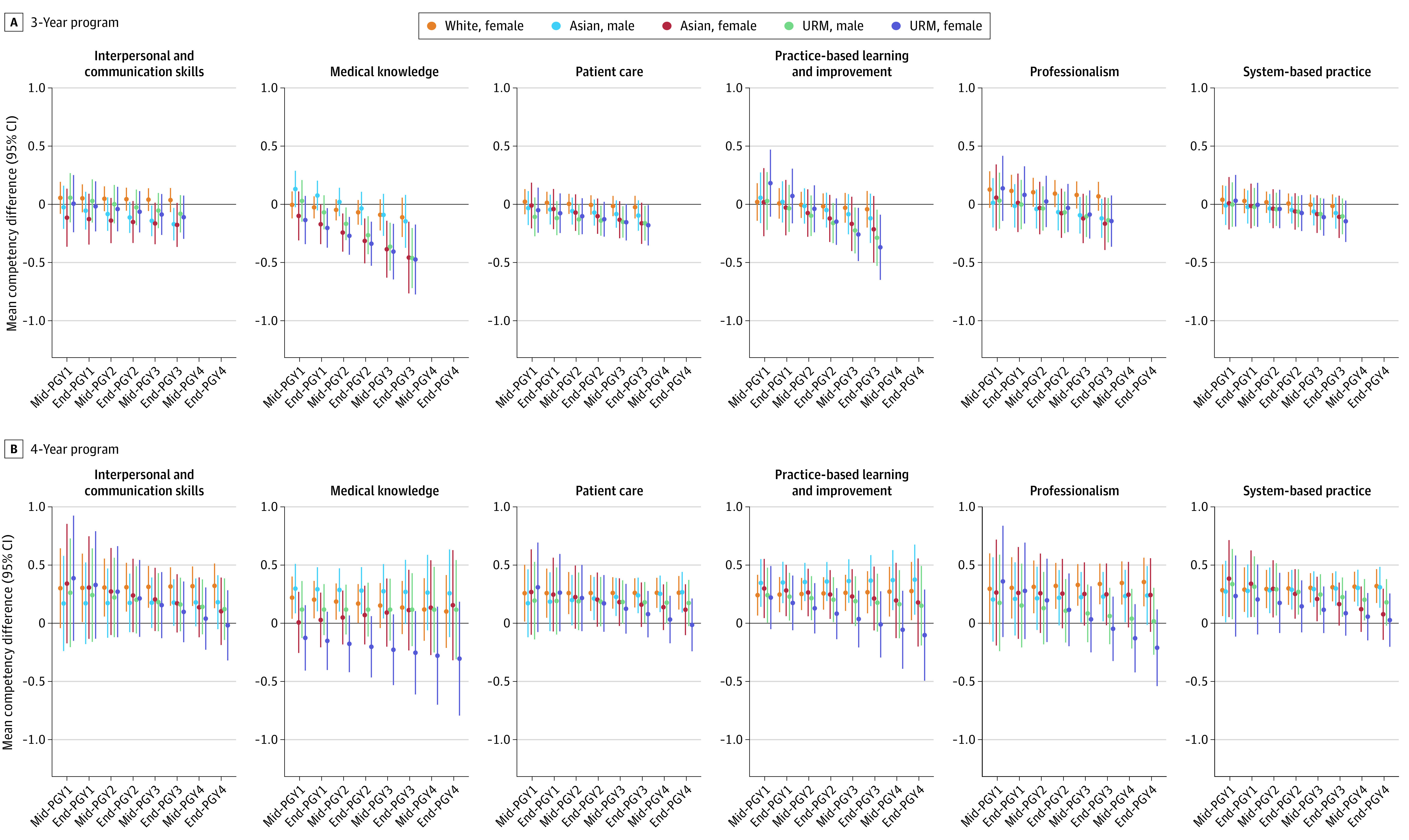
Intersectional Disparities in Competency Scores by Ethnoracial Group and Sex Cross-Strata A, Linear contrasts and corresponding 95% CIs comparing each ethnoracial group and sex cross-strata to White male residents in 3-y programs. B, 4-y programs for each postgraduate year (PGY) time point. Mixed-effects models included a random intercept for individual residents and programs as well as a random slope for time. The model also adjusted for United States Medical Licensing Examination Step 2 Clinical Knowledge Step 2 CK scores as fixed effects. URM indicates underrepresented in medicine.

In adjusted models, except for among White female residents, we observed worsening disparities in milestone scores for minoritized EM residents (Figure 3; eTables 7 and 8 in Supplement 1). Similar to the unadjusted estimates at PGY1 midyear, URM male and female residents in 3-year programs were initially rated comparably with White male residents; however, they were rated increasingly lower than White male residents in 3 of 6 competencies by PGY3 year-end. This included significantly lower scores in these domains: medical knowledge (URM male residents, −0.46 [95% CI, −0.72 to −0.20]; URM female residents, −0.47 [95% CI, −0.77 to −0.17]), patient care (URM male residents, −0.16 [95% CI, −0.31 to −0.01]; URM female residents, −0.18 [95% CI, −0.35 to −0.01]), and practice-based learning and improvement (URM male residents, −0.29 [95% CI, −0.53 to −0.05]; URM female residents, −0.37 [95% CI, −0.65 to −0.09]). Similar patterns were observed for URM female residents in 4-year programs across all competencies to a greater degree, but this was not observed for URM male residents. We also noted differential trends between 3- and 4-year programs for Asian EM residents. Asian male residents in 3-year programs received significantly lower scores in the interpersonal and communication skills domain than White male residents by PGY3 year-end (−0.17; 95% CI, −0.31 to −0.03), yet there were no differences over the assessment period in 4-year programs. Asian female residents in 3-year programs experienced increasing gaps in medical knowledge scores by PGY3 year-end (−0.46; 95% CI, −0.77 to −0.15), whereas in 4-year programs, the disparity in these scores narrowed for Asian female residents from PGY1 midyear (−0.29; 95% CI, −0.55 to −0.03) to PGY4 year-end (−0.14; 95% CI, −0.62 to 0.33).

### Overall Assessment Scores

**Figure 4.**
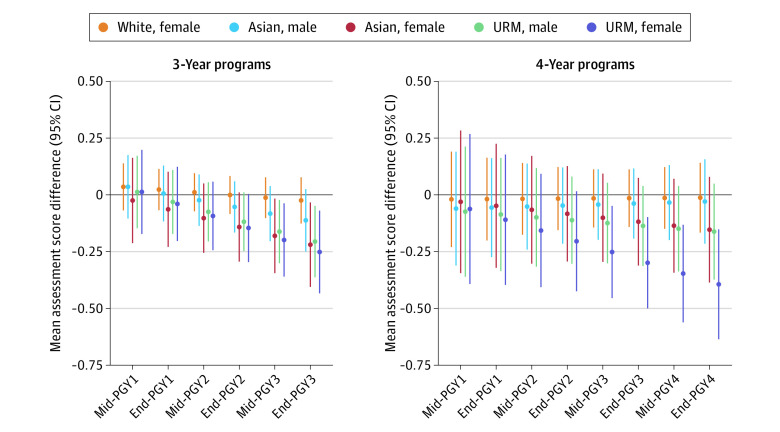
Association of Intersectional Disparities on Assessment Score Trajectory Linear contrasts and corresponding 95% CIs of each ethnoracial group and sex cross-strata compared with White male residents in 3-y and 4-y programs for each postgraduate year (PGY) time point. Mixed-effects models included a random intercept for individual residents and programs as well as a random slope for time. The model also adjusted for United States Medical Licensing Examination Step 2 Clinical Knowledge scores as fixed effects. URM indicates underrepresented in medicine.

Consistent with competency domain scores, the mean overall Milestone scores displayed similar patterns at PGY1 midyear and throughout the assessment period (eFigure 3 in Supplement 1). We also observed comparable trends in the total scores across ethnoracial group−sex cross-strata. Among residents in 3-year programs, scores were similar between ethnoracial group−sex at PGY1 midyear, but in 4-year programs, only URM female residents (mean [SD], 2.29 [0.90]) received higher scores than White male residents (mean [SD], 2.01 [0.72]) (eFigure 4 and eTable 9 in Supplement 1). Moreover, all minoritized residents, except for White female residents, experienced progressively lower ratings by PGY2 midyear, regardless of whether they were in 3- or 4-year programs. After model adjustment, we observed no differences in the overall Milestone scores between minoritized and White male residents from PGY1 midyear to PGY2 midyear in both 3- and 4-year programs (Figure 4; eTable 10 in Supplement 1). However, by PGY3 year-end, Asian female residents (−0.22; 95% CI, −0.41 to −0.03), URM male residents (−0.21; 95% CI, −0.45 to −0.05), and URM female residents (−0.25; 95% CI, −0.43 to −0.07) in 3-year programs received lower ratings than White male residents. In 4-year programs, only URM female residents were rated increasingly lower than White male residents from PGY3 midyear (−0.25; 95% CI, −0.45 to −0.05) to PGY4 year-end (−0.39; 95% CI, −0.64 to −0.15). Similar trends were not observed for White female residents in either program length and to a limited extent for Asian male residents in 3-year programs and Asian female residents and URM male residents in 4-year programs.

## Discussion

Our study quantified intersectional sex-specific ethnoracial disparities in Milestone assessments among EM residents. Compared with White male residents, URM female residents consistently received lower assessment scores with disparate rating gaps increasing over time. In 3-year programs, URM female residents were evaluated approximately 0.25 points lower than White male residents, which equated to approximately 2 months of additional training based on an average annual increase in milestone scores of 1.47 points. The disparities were even more pronounced for URM female residents in 4-year programs where the difference of 0.39 milestone points was equivalent to 3 additional months of training, considering an annual increase of 1.28 points. Additionally, we found that URM female residents in 4-year programs received increasingly lower assessment scores for all competency domains, which was only exhibited in 3 of 6 competencies in 3-year programs. The URM male residents had similarly disparate trends in assessments although the gap with respect to White male residents was blunted. In comparison, assessments for Asian male and female residents were significantly lower in only a subset of competencies (*interpersonal communication skills* and *medical knowledge*) at later assessment windows, and in specific, with program lengths. Notably, there were no statistically significant differences in assessments between White male and female residents in our analyses in either 3- or 4-year programs. Overall, disparities were more pronounced in 4-year programs in comparison with 3-year programs, which we attribute to the increased duration of exposure to factors such as bias and discrimination that may contribute to assessment score differences across groups.

Previous studies of disparities in resident assessments have focused on gendered^19,21^ and/or racial disparities in isolation,^36^ with more emphasis on the former. Our study improves on the prior literature by applying an intersectional framework to the investigation of EM resident assessment disparities. Under this framework, and in practice, the manifestations of ethnoracial and sex bias that may underlie these assessment disparities are inextricably linked; URM female residents are not subject to discrimination on the basis of their sex separate from their race or ethnicity. Through our analyses, we found that URM female residents compared with URM male and White female residents, experienced the most severe disparities; this is a novel finding that would have been obscured by a nonintersectional design. In a previous study of 8 EM residency programs, Dayal and colleagues^21^ found that male residents scored approximately 0.15 Milestone levels higher than female residents, which they equated to 3 to 4 months of training.^21^ However, this result was not replicated in a larger follow-up study,^23^ consistent with our intersectional analyses that did not show a statistically significant difference between White male and White female assessment scores. This suggests that the gender disparities observed by Dayal and colleagues^21^ may have been driven by *gendered racial disparities*, specific to female residents subject to the interaction of racism and sexism. This distinction highlights the importance of intersectional studies in accurately describing the effects of discrimination.

In comparison, studies on racial disparities within residency programs have focused on experiences of interpersonal racism rather than estimating inequities in standardized performance assessments.^36,37,38^ These studies demonstrated that URM trainees experience more discrimination than their peers, and that this is often on the basis of their race. However, these studies generally reported experiences across race and gender strata separately and did not specifically evaluate the experiences of those at the intersections (ie, URM female residents). To our knowledge, this is the first study to evaluate racial disparities in performance assessments, done so with the additional theoretical rigor of intersectionality. Beyond these conceptual improvements, our longitudinal study of EM residents in more than 120 programs provides stronger and more generalizable evidence of resident assessment disparities than previous studies with a cross-sectional design^19^ or with smaller sample sizes.^21^

Discrimination in the clinical learning environment affects retention and promotion of female, URM, and URM female physicians, who are critical for diversifying the health care workforce. Importantly, potential sex-specific ethnoracial discrimination in the form of assessment disparities, may also directly affect the psychosocial well being of minoritized trainees. Several studies have demonstrated an association between experiences of discrimination and mistreatment with attrition,^39^ burnout,^40,41^ and depression^42^ among medical students and physicians who are female and/or from minoritized ethnoracial backgrounds. These studies indicate that bias and discrimination may function as root causes^43^ leading to adverse health outcomes for URM, female, and URM female trainees. As such, eliminating the sex-specific ethnoracial in assessments as demonstrated in this study may contribute to maintaining the emotional and mental health of trainees and potentially improve patient health through a positive feedback mechanism that includes physician well-being, retention, and promotion of minoritized trainees, and more equitable health care provision.

### Limitations

This study has limitations. Demographic data are an imperfect proxy of exposure to systemic discrimination,^32^ and intersectional sex-specific ethnoracial disparities in assessments only capture a subset of the total discrimination that URM, female, and URM female residents may experience during training. Additionally, this study only had access to binary sex data, which incompletely captures the full spectrum of gender identity and does not allow the disaggregation of transgender and nonbinary trainees. This limitation precludes the observation of any disparities in resident assessments among transgender and nonbinary EM residents, despite evidence that experiences of discrimination among this group are high.^44^

Notably, we excluded nearly one-third of EM programs because they did not include at least 1 URM and 1 Asian trainee during the study period (eFigure 1 in Supplement 1). This limitation reflects the absence of ethnoracial representation in EM^45,46^ and highlights the continued need for diversifying the health care workforce, consistent with the ACGME accreditation standards across all specialties that are URM.^47^ Additionally, our exclusion of international medical school graduates limits the generalizability of our results and precludes our ability to robustly assess the contribution of xenophobia to disparities in EM resident assessments.

## Conclusions

This retrospective cohort study of ACGME Milestone assessments for 2708 EM residents in 128 programs found significant evidence of sex-specific racial and ethnic disparities in performance assessments throughout the training period. These disparities were most severe for URM female and Asian female residents, followed by URM male compared with White male residents. Eliminating sex-specific ethnoracial disparities in resident assessments may contribute to equitable health care by removing barriers to retention and promotion of underrepresented and minoritized trainees, thereby facilitating the diversity of the emergency physician workforce.

Achieving health equity for minoritized ethnoracial groups and other marginalized populations is an urgent public health priority. Escalating public attention on long-standing health inequities that disproportionately affect Black, Indigenous, Latine, and low-income communities, and the intersections of these in the US has amplified our collective commitment to improving representation in health care as a lever for catalyzing health justice.

**Funding/Support: **This study was supported through grants to Dr Boatright from the US National Institutes of Health (No. R21MD013481) and the Emergency Medicine Foundation.

## Notes

Supplement 1. **eTable 1.** Resident Characteristics (AAMC) and Evaluations (ACGME) Data Merge **eTable 2.** ACGME Emergency Resident Milestones **eTable 3.** Residents Per Academic Year, Per Training Year **eTable 4.** Residents Per Evaluation Period **eTable 5.** Mean Competency Scores Over the Evaluation Period for US Emergency Residents in Three-Year Programs by Each Ethnoracial Group and Sex Cross-Strata **eTable 6.** Mean Competency Scores Over the Evaluation Period for US Emergency Residents in Four-Year Programs by Each Ethnoracial Group and Sex Cross-Strata **eTable 7.** Adjusted Differences in Mean Competency Scores Over the Evaluation Period for US Emergency Residents in Three-Year Programs by Each Ethnoracial Group and Sex Cross-Strata **eTable 8.** Adjusted Differences in Mean Competency Scores Over the Evaluation Period for US Emergency Residents in Four-Year Programs by Each Ethnoracial Group and Sex Cross-Strata **eTable 9.** Mean Evaluation Scores for Three-Year and Four-Year Programs Over the Study Period by Each Ethnoracial Group and Sex Cross-Strata **eTable 10.** Adjusted Differences in Mean Evaluation Scores for Three-Year and Four-Year Programs Over the Study Period by Each Ethnoracial Group and Sex Cross-Strata **eFigure 1.** Analytic Cohort Development and Exclusion Criteria **eFigure 2.** Mean Competency Scores for the Overall Sample **eFigure 3.** Mean Assessment Score for the Overall Sample **eFigure 4.** Assessment Score Trajectories by Ethnoracial Group and Sex Cross-Strata

Supplement 2.
Data Sharing Statement
