# Supplementary material for: Intersectional Disparities in Emergency Medicine Residents’ Performance Assessments by Race, Ethnicity, and Sex
Source: JAMA Netw Open. 2023 Sep 21;6(9):e2330847. doi: 10.1001/jamanetworkopen.2023.30847 (PMC10514741; doi:10.1001/jamanetworkopen.2023.30847)
Supplement: Supplement 1. — eTable 1. Resident Characteristics (AAMC) and Evaluations (ACGME) Data Merge eTable 2. ACGME Emergency Resident Milestones eTable 3. Residents Per Academic Year, Per Training Year eTable 4. Residents Per Evaluation Period eTable 5. Mean Competency Scores Over the Evaluation Period for US Emergency Residents in Three-Year Programs by Each Ethnoracial Group and Sex Cross-Strata eTable 6. Mean Competency Scores Over the Evaluation Period for US Emergency Residents in Four-Year Programs by Each Ethnoracial Group and Sex Cross-Strata eTable 7. Adjusted Differences in Mean Competency Scores Over the Evaluation Period for US Emergency Residents in Three-Year Programs by Each Ethnoracial Group and Sex Cross-Strata eTable 8. Adjusted Differences in Mean Competency Scores Over the Evaluation Period for US Emergency Residents in Four-Year Programs by Each Ethnoracial Group and Sex Cross-Strata eTable 9. Mean Evaluation Scores for Three-Year and Four-Year Programs Over the Study Period by Each Ethnoracial Group and Sex Cross-Strata eTable 10. Adjusted Differences in Mean Evaluation Scores for Three-Year and Four-Year Programs Over the Study Period by Each Ethnoracial Group and Sex Cross-Strata eFigure 1. Analytic Cohort Development and Exclusion Criteria eFigure 2. Mean Competency Scores for the Overall Sample eFigure 3. Mean Assessment Score for the Overall Sample eFigure 4. Assessment Score Trajectories by Ethnoracial Group and Sex Cross-Strata [file jamanetwopen-e2330847-s001.pdf]

Supplemental Online Content

Lett E, Tran NK, Nweke N, et al. Intersectional disparities in emergency medicine residents' performance assessments by race, ethnicity, and sex. *JAMA Netw Open.* 2023;6(8):e2330847. doi:10.1001/jamanetworkopen.2023.30847

eTable 1. Resident Characteristics (AAMC) and Evaluations (ACGME) Data Merge

eTable 2. ACGME Emergency Resident Milestones

eTable 3. Residents Per Academic Year, Per Training Year

eTable 4. Residents Per Evaluation Period

eTable 5. Mean Competency Scores Over the Evaluation Period for US Emergency Residents in Three-Year Programs by Each Ethnoracial Group and Sex Cross-Strata

eTable 6. Mean Competency Scores Over the Evaluation Period for US Emergency Residents in Four-Year Programs by Each Ethnoracial Group and Sex Cross-Strata

eTable 7. Adjusted Differences in Mean Competency Scores Over the Evaluation Period for US Emergency Residents in Three-Year Programs by Each Ethnoracial Group and Sex Cross-Strata

eTable 8. Adjusted Differences in Mean Competency Scores Over the Evaluation Period for US Emergency Residents in Four-Year Programs by Each Ethnoracial Group and Sex Cross-Strata

eTable 9. Mean Evaluation Scores for Three-Year and Four-Year Programs Over the Study Period by Each Ethnoracial Group and Sex Cross-Strata

eTable 10. Adjusted Differences in Mean Evaluation Scores for Three-Year and Four-Year Programs Over the Study Period by Each Ethnoracial Group and Sex Cross-Strata

eFigure 1. Analytic Cohort Development and Exclusion Criteria

eFigure 2. Mean Competency Scores for the Overall Sample

eFigure 3. Mean Assessment Score for the Overall Sample

eFigure 4. Assessment Score Trajectories by Ethnoracial Group and Sex Cross-Strata

This supplemental material has been provided by the authors to give readers additional information about their work.

eTable 1. Resident Characteristics (AAMC) and Evaluations (ACGME) Data Merge

	Resident Characteristics Data	Evaluations Data	Union (Present in either or both datasets)	Intersection (present in both datasets)
Number of Residents (Number of Programs)	4368 (209 programs)	4506 (216 programs)	4591 (216 total programs)	4283 (209 Programs)
Numbers are not present in other data source	85	223	308	0

© 2023 Lett E et al. *JAMA Netw Open.*

eTable 2. ACGME Emergency Resident Milestones

Competency	Subcompetency
Interpersonal and Communication Skills	ICS1: Patient-Centered Communication
	ICS2: Team Management
Medical Knowledge	MK: Medical Knowledge
Patient Care	PC1: Emergency Stabilization
	PC2: Performance of Focused History and Physical Exam
	PC3: Diagnostic Studies
	PC4: Diagnosis
	PC5: Pharmacotherapy
	PC6: Observation and Reassessment
	PC7: Disposition
	PC8: Multi-tasking (Task-switching)
	PC9: General Approach to Procedures
	PC10: Airway Management
	PC11: Anesthesia and Acute Pain Management
	PC12: Other Diagnostic and Therapeutic Procedures: Goal-directed Focused Ultrasound
	PC13: Other Diagnostic and Therapeutic Procedures: Wound Management
	PC14: Other Diagnostic and Therapeutic Procedures: Vascular Access
Practice-Based Learning and Improvement	PBLI: Practice-Based Performance Improvement
Professionalism	PROF1: Professional Values
	PROF2: Accountability
Systems-Based Practice	SBP1: Patient Safety
	SBP2: Systems-based Management
	SBP3: Technology

© 2023 Lett E et al. *JAMA Netw Open.*

eTable 3. Residents Per Academic Year, Per Training Year

Residents in Three-Year Programs				
Academic Year	PGY-1	PGY-2	PGY-3	PGY-4
2014-2015	937	–	–	–
2015-2016	938	954	–	–
2016-2017	–	934	947	–
2017-2018	–	–	947	–
**Residents in Four-Year Programs**				
Academic Year	PGY-1	PGY-2	PGY-3	PGY-4
2014-2015	387	388	–	–
2015-2016	–	386	385	–
2016-2017	–	–	388	395
2017-2018	–	–	–	389

© 2023 Lett E et al. *JAMA Netw Open.*

eTable 4. Residents Per Evaluation Period

Residency Year	Evaluation Point	Total	Three-Year	Four-Year
First	Mid-Year	2260	1873	387
	Year-End	2245	1858	387
Second	Mid-Year	2644	1873	771
	Year-End	2642	1869	773
Third	Mid-Year	2652	1880	772
	Year-End	2639	1867	772
Fourth	Mid-Year	784	–	784
	Year-End	768	–	768

© 2023 Lett E et al. *JAMA Netw Open.*

eTable 5. Mean Competency Scores Over the Evaluation Period for US Emergency Residents in *Three-Year* Programs by Each Ethnoracial Group and Sex Cross-Strata

Assessment Period	Racial Groups	Sex	Interpersonal and Communication Skills Mean (SD)	Medical Knowledge Mean (SD)	Patient Care Mean (SD)	Practice-Based Learning and Improvement Mean (SD)	Professionalism Mean (SD)	System-Based Practice Mean (SD)
Mid-PGY1	White	Male	2.55 (0.92)	1.91 (0.88)	2.35 (0.72)	2.39 (1.03)	2.61 (0.95)	2.39 (0.83)
Mid-PGY1	White	Female	2.66 (0.96)	1.92 (0.87)	2.44 (0.72)	2.44 (1.00)	2.77 (1.02)	2.48 (0.84)
Mid-PGY1	Asian	Male	2.52 (0.96)	1.95 (0.98)	2.28 (0.78)	2.43 (1.05)	2.60 (1.11)	2.33 (0.88)
Mid-PGY1	Asian	Female	2.54 (0.94)	1.94 (1.06)	2.40 (0.77)	2.45 (1.15)	2.73 (1.01)	2.50 (0.86)
Mid-PGY1	URM	Male	2.55 (1.10)	1.74 (0.88)	2.18 (0.78)	2.30 (1.21)	2.63 (1.23)	2.24 (0.98)
Mid-PGY1	URM	Female	2.60 (1.01)	1.89 (0.88)	2.34 (0.75)	2.57 (1.16)	2.76 (1.03)	2.48 (0.95)
End-PGY1	White	Male	3.48 (0.95)	3.15 (1.21)	3.38 (0.76)	3.32 (1.01)	3.47 (0.95)	3.36 (0.85)
End-PGY1	White	Female	3.57 (0.94)	3.15 (1.16)	3.44 (0.76)	3.47 (0.95)	3.61 (1.00)	3.47 (0.86)
End-PGY1	Asian	Male	3.39 (1.01)	3.18 (1.29)	3.27 (0.77)	3.40 (1.07)	3.43 (1.00)	3.32 (0.86)
End-PGY1	Asian	Female	3.30 (0.88)	2.9 (1.19)	3.32 (0.75)	3.37 (0.97)	3.46 (1.00)	3.37 (0.89)
End-PGY1	URM	Male	3.42 (1.12)	2.94 (1.24)	3.17 (0.83)	3.34 (1.09)	3.48 (1.12)	3.32 (1.09)
End-PGY1	URM	Female	3.53 (1.03)	2.75 (1.12)	3.25 (0.80)	3.55 (1.08)	3.57 (1.12)	3.44 (0.90)
Mid-PGY2	White	Male	4.58 (0.93)	4.02 (1.22)	4.58 (0.74)	4.33 (0.96)	4.50 (0.96)	4.47 (0.83)
Mid-PGY2	White	Female	4.64 (0.88)	4.00 (1.19)	4.61 (0.72)	4.41 (0.85)	4.59 (0.98)	4.50 (0.82)
Mid-PGY2	Asian	Male	4.37 (1.02)	4.01 (1.29)	4.43 (0.86)	4.24 (1.07)	4.43 (1.05)	4.32 (0.92)
Mid-PGY2	Asian	Female	4.33 (1.02)	3.74 (1.18)	4.37 (0.78)	4.28 (0.92)	4.44 (0.94)	4.26 (0.86)
Mid-PGY2	URM	Male	4.51 (1.05)	3.71 (1.21)	4.36 (0.77)	4.20 (0.95)	4.37 (0.98)	4.32 (0.92)
Mid-PGY2	URM	Female	4.43 (0.88)	3.52 (1.16)	4.30 (0.77)	4.31 (0.93)	4.35 (0.98)	4.32 (0.86)
End-PGY2	White	Male	5.30 (0.86)	5.12 (1.22)	5.33 (0.70)	5.03 (0.92)	5.22 (0.92)	5.21 (0.75)
End-PGY2	White	Female	5.40 (0.81)	5.01 (1.20)	5.33 (0.67)	5.01 (1.01)	5.27 (0.97)	5.23 (0.72)
End-PGY2	Asian	Male	5.11 (0.90)	4.94 (1.26)	5.21 (0.76)	4.89 (1.05)	5.07 (0.98)	5.06 (0.80)
End-PGY2	Asian	Female	5.07 (0.91)	4.81 (1.24)	5.06 (0.81)	4.93 (1.06)	4.97 (1.15)	5.06 (0.82)
End-PGY2	URM	Male	5.18 (0.90)	4.68 (1.29)	5.07 (0.74)	4.88 (1.00)	5.11 (1.05)	5.01 (0.86)
End-PGY2	URM	Female	5.27 (0.82)	4.64 (1.31)	5.06 (0.70)	4.83 (1.12)	5.08 (0.95)	5.10 (0.82)
Mid-PGY3	White	Male	6.23 (0.85)	5.78 (1.18)	6.29 (0.70)	5.92 (1.10)	6.02 (0.98)	6.13 (0.76)
Mid-PGY3	White	Female	6.34 (0.72)	5.66 (1.24)	6.36 (0.62)	6.01 (0.92)	6.14 (0.89)	6.18 (0.69)
Mid-PGY3	Asian	Male	6.05 (0.93)	5.60 (1.23)	6.19 (0.73)	5.87 (1.20)	5.93 (1.03)	6.09 (0.75)
Mid-PGY3	Asian	Female	6.05 (0.97)	5.35 (1.31)	6.18 (0.77)	5.79 (1.10)	5.91 (1.01)	6.05 (0.84)
Mid-PGY3	URM	Male	6.09 (0.87)	5.36 (1.26)	6.05 (0.74)	5.64 (1.12)	5.90 (1.06)	5.89 (0.76)
Mid-PGY3	URM	Female	6.19 (0.73)	5.40 (1.28)	6.14 (0.66)	5.81 (0.85)	6.04 (0.98)	6.08 (0.72)

© 2023 Lett E et al. *JAMA Netw Open.*

End-PGY3	White	Male	6.93 (0.67)	6.66 (1.06)	7.01 (0.56)	6.79 (0.96)	6.80 (0.89)	6.87 (0.61)
End-PGY3	White	Female	7.00 (0.61)	6.61 (1.03)	7.04 (0.49)	6.81 (0.79)	6.90 (0.76)	6.92 (0.56)
End-PGY3	Asian	Male	6.80 (0.74)	6.48 (1.06)	6.89 (0.55)	6.71 (0.98)	6.69 (0.89)	6.76 (0.63)
End-PGY3	Asian	Female	6.85 (0.69)	6.31 (1.15)	6.89 (0.58)	6.65 (1.05)	6.74 (0.75)	6.84 (0.59)
End-PGY3	URM	Male	6.80 (0.89)	5.94 (1.45)	6.82 (0.64)	6.47 (1.18)	6.64 (1.09)	6.71 (0.69)
End-PGY3	URM	Female	6.88 (0.65)	6.17 (1.35)	6.86 (0.55)	6.48 (1.39)	6.62 (1.04)	6.81 (0.64)

© 2023 Lett E et al. *JAMA Netw Open.*

eTable 6. Mean Competency Scores Over the Evaluation Period for US Emergency Residents in *Four-Year Programs* by Each Ethnoracial Group and Sex Cross-Strata

Assessment Period	Racial Groups	Sex	Interpersonal and Communication Skills	Medical Knowledge	Patient Care	Practice-Based Learning and Improvement	Professionalism	System-Based Practice
			Mean (SD)	Mean (SD)	Mean (SD)	Mean (SD)	Mean (SD)	Mean (SD)
Mid-PGY1	White	Male	2.29 (1.04)	1.43 (0.68)	2.03 (0.76)	1.90 (0.90)	2.40 (1.07)	1.98 (0.78)
Mid-PGY1	White	Female	2.37 (1.09)	1.36 (0.55)	2.04 (0.71)	2.02 (1.01)	2.30 (0.95)	1.99 (0.77)
Mid-PGY1	Asian	Male	2.29 (1.22)	1.55 (0.75)	1.99 (0.80)	1.93 (0.99)	2.35 (1.01)	2.01 (0.91)
Mid-PGY1	Asian	Female	2.34 (1.30)	1.41 (0.61)	2.07 (0.75)	2.00 (1.02)	2.24 (1.08)	2.11 (0.90)
Mid-PGY1	URM	Male	2.22 (0.97)	1.43 (0.78)	2.04 (0.72)	1.90 (0.90)	2.21 (1.17)	2.03 (0.84)
Mid-PGY1	URM	Female	2.77 (1.48)	1.54 (0.69)	2.20 (0.95)	2.25 (0.97)	2.75 (1.00)	2.25 (0.99)
End-PGY1	White	Male	3.21 (0.84)	2.64 (0.92)	2.93 (0.54)	2.61 (0.82)	3.15 (0.83)	2.91 (0.77)
End-PGY1	White	Female	3.17 (0.92)	2.49 (0.78)	2.86 (0.64)	2.69 (0.92)	3.09 (0.89)	2.83 (0.77)
End-PGY1	Asian	Male	3.22 (0.84)	2.64 (0.74)	2.87 (0.57)	2.66 (0.85)	3.12 (0.69)	2.89 (0.73)
End-PGY1	Asian	Female	3.10 (1.13)	2.24 (0.65)	2.79 (0.56)	2.62 (1.28)	3.01 (0.78)	2.96 (0.76)
End-PGY1	URM	Male	2.92 (0.90)	2.25 (0.67)	2.74 (0.55)	2.28 (0.75)	2.86 (0.85)	2.76 (0.65)
End-PGY1	URM	Female	3.30 (0.91)	2.32 (0.82)	2.97 (0.52)	2.82 (0.86)	3.29 (0.84)	3.02 (0.80)
Mid-PGY2	White	Male	4.03 (0.96)	3.32 (1.03)	3.92 (0.71)	3.72 (0.90)	3.93 (0.93)	3.81 (0.77)
Mid-PGY2	White	Female	4.08 (0.90)	3.21 (0.92)	3.84 (0.71)	3.60 (0.93)	3.97 (0.88)	3.79 (0.79)
Mid-PGY2	Asian	Male	4.02 (0.98)	3.23 (0.91)	3.85 (0.71)	3.75 (0.97)	3.97 (0.88)	3.83 (0.87)
Mid-PGY2	Asian	Female	3.98 (1.08)	2.98 (1.08)	3.84 (0.79)	3.55 (0.98)	3.83 (0.97)	3.76 (0.90)
Mid-PGY2	URM	Male	3.82 (1.24)	2.82 (0.84)	3.64 (0.79)	3.56 (1.01)	3.69 (1.19)	3.66 (0.94)
Mid-PGY2	URM	Female	3.96 (0.92)	2.96 (0.83)	3.78 (0.70)	3.47 (0.93)	3.88 (1.00)	3.60 (0.79)
End-PGY2	White	Male	4.78 (0.89)	4.49 (1.10)	4.66 (0.70)	4.52 (0.90)	4.67 (0.87)	4.54 (0.74)
End-PGY2	White	Female	4.68 (0.97)	4.31 (1.16)	4.56 (0.75)	4.37 (1.00)	4.59 (0.99)	4.49 (0.88)
End-PGY2	Asian	Male	4.60 (0.90)	4.26 (1.02)	4.56 (0.73)	4.55 (0.93)	4.59 (0.81)	4.55 (0.90)
End-PGY2	Asian	Female	4.69 (1.15)	4.36 (1.02)	4.51 (0.82)	4.59 (0.90)	4.59 (1.09)	4.54 (0.90)
End-PGY2	URM	Male	4.57 (1.19)	4.12 (1.03)	4.42 (0.78)	4.30 (0.89)	4.36 (1.26)	4.51 (0.99)
End-PGY2	URM	Female	4.68 (0.87)	3.72 (1.03)	4.52 (0.78)	4.26 (0.90)	4.42 (1.13)	4.39 (0.85)
Mid-PGY3	White	Male	5.58 (0.84)	5.04 (1.18)	5.57 (0.64)	5.22 (1.01)	5.43 (0.88)	5.41 (0.69)
Mid-PGY3	White	Female	5.61 (0.83)	4.89 (1.23)	5.50 (0.68)	5.18 (0.96)	5.41 (0.99)	5.43 (0.75)
Mid-PGY3	Asian	Male	5.46 (0.88)	4.92 (1.21)	5.52 (0.67)	5.39 (0.90)	5.45 (0.87)	5.52 (0.87)
Mid-PGY3	Asian	Female	5.55 (0.98)	4.93 (1.06)	5.41 (0.77)	5.20 (0.96)	5.38 (0.91)	5.36 (0.80)
Mid-PGY3	URM	Male	5.50 (1.05)	4.89 (1.24)	5.46 (0.74)	5.30 (1.05)	5.21 (1.15)	5.43 (0.80)
Mid-PGY3	URM	Female	5.57 (0.85)	4.40 (1.08)	5.51 (0.68)	5.08 (0.90)	5.24 (1.26)	5.37 (0.68)

End-PGY3	White	Male	6.15 (0.79)	5.76 (1.21)	6.20 (0.61)	5.91 (0.93)	5.97 (0.86)	5.97 (0.66)
End-PGY3	White	Female	6.22 (0.80)	5.43 (1.34)	6.17 (0.61)	5.86 (0.88)	6.01 (0.86)	6.03 (0.70)
End-PGY3	Asian	Male	6.04 (0.79)	5.50 (1.26)	6.13 (0.68)	5.91 (0.86)	5.93 (0.84)	6.05 (0.79)
End-PGY3	Asian	Female	6.17 (0.81)	5.63 (1.05)	6.18 (0.55)	5.82 (0.87)	6.06 (0.81)	5.95 (0.63)
End-PGY3	URM	Male	6.07 (0.88)	5.27 (1.16)	6.00 (0.68)	5.75 (0.97)	5.80 (0.86)	5.89 (0.64)
End-PGY3	URM	Female	5.86 (0.98)	5.08 (1.16)	5.96 (0.72)	5.45 (0.99)	5.59 (1.11)	5.81 (0.70)
Mid-PGY4	White	Male	6.90 (0.83)	6.31 (1.2)	6.93 (0.62)	6.61 (1.15)	6.75 (0.86)	6.72 (0.67)
Mid-PGY4	White	Female	6.90 (0.65)	6.05 (1.28)	6.88 (0.53)	6.59 (0.89)	6.79 (0.72)	6.73 (0.56)
Mid-PGY4	Asian	Male	6.71 (0.73)	6.26 (1.12)	6.89 (0.48)	6.64 (0.74)	6.70 (0.71)	6.72 (0.60)
Mid-PGY4	Asian	Female	6.69 (0.64)	6.15 (1.10)	6.78 (0.54)	6.54 (0.86)	6.70 (0.82)	6.55 (0.58)
Mid-PGY4	URM	Male	6.72 (0.74)	6.00 (1.15)	6.78 (0.62)	6.46 (1.14)	6.59 (0.93)	6.62 (0.64)
Mid-PGY4	URM	Female	6.51 (1.18)	5.73 (1.45)	6.60 (1.06)	6.29 (1.25)	6.43 (1.27)	6.44 (1.07)
End-PGY4	White	Male	7.32 (0.62)	6.89 (0.88)	7.40 (0.48)	7.14 (0.85)	7.22 (0.62)	7.21 (0.47)
End-PGY4	White	Female	7.33 (0.57)	6.78 (1.00)	7.36 (0.43)	7.17 (0.62)	7.22 (0.57)	7.17 (0.46)
End-PGY4	Asian	Male	7.23 (0.61)	6.82 (1.08)	7.36 (0.42)	7.15 (0.57)	7.19 (0.61)	7.19 (0.49)
End-PGY4	Asian	Female	7.17 (0.54)	6.77 (0.95)	7.29 (0.44)	7.05 (0.72)	7.16 (0.65)	7.03 (0.42)
End-PGY4	URM	Male	7.03 (0.59)	6.63 (1.02)	7.18 (0.48)	6.87 (1.03)	6.87 (0.82)	7.02 (0.43)
End-PGY4	URM	Female	7.02 (0.71)	6.65 (0.89)	7.13 (0.53)	6.94 (0.73)	6.84 (0.89)	6.95 (0.57)

© 2023 Lett E et al. *JAMA Netw Open.*

eTable 7. Adjusted Differences in Mean Competency Scores Over the Evaluation Period for US Emergency Residents in *Three-Year Programs* by Each Ethnoracial Group and Sex Cross-Strata

Assessment Period	Racial Groups	Sex	Interpersonal and Communication Skills		Medical Knowledge		Patient Care		Practice-Based Learning and Improvement		Professionalism		System-Based Practice	
			β	95% CI	β	95% CI	β	95% CI	β	95% CI	β	95% CI	β	95% CI
Mid-PGY1	White	Female	0.055	-0.082, 0.192	-0.005	-0.121, 0.111	0.022	-0.086, 0.130	0.020	-0.141, 0.181	0.127	-0.030, 0.284	0.039	-0.085, 0.163
Mid-PGY1	Asian	Male	-0.026	-0.211, 0.159	0.131	-0.026, 0.288	-0.032	-0.178, 0.114	0.056	-0.161, 0.274	0.013	-0.198, 0.225	-0.010	-0.177, 0.157
Mid-PGY1	Asian	Female	-0.115	-0.364, 0.135	-0.100	-0.309, 0.110	-0.010	-0.207, 0.186	0.018	-0.274, 0.310	0.058	-0.227, 0.342	0.009	-0.216, 0.234
Mid-PGY1	URM	Male	0.056	-0.155, 0.268	0.029	-0.150, 0.208	-0.108	-0.275, 0.059	0.028	-0.220, 0.276	0.031	-0.209, 0.272	-0.002	-0.193, 0.189
Mid-PGY1	URM	Female	0.006	-0.240, 0.251	-0.135	-0.342, 0.072	-0.051	-0.244, 0.143	0.182	-0.106, 0.470	0.137	-0.142, 0.416	0.031	-0.191, 0.252
End-PGY1	White	Female	0.051	-0.068, 0.171	-0.026	-0.121, 0.068	0.013	-0.082, 0.108	0.008	-0.123, 0.138	0.115	-0.023, 0.253	0.029	-0.077, 0.134
End-PGY1	Asian	Male	-0.055	-0.216, 0.107	0.076	-0.052, 0.203	-0.045	-0.173, 0.083	0.021	-0.156, 0.198	-0.013	-0.200, 0.173	-0.023	-0.165, 0.119
End-PGY1	Asian	Female	-0.127	-0.345, 0.091	-0.171	-0.342, 0.000	-0.041	-0.213, 0.131	-0.028	-0.266, 0.209	0.013	-0.238, 0.263	-0.014	-0.206, 0.177
End-PGY1	URM	Male	0.029	-0.156, 0.214	-0.069	-0.216, 0.077	-0.119	-0.265, 0.028	-0.035	-0.237, 0.168	-0.002	-0.214, 0.210	-0.022	-0.185, 0.140
End-PGY1	URM	Female	-0.018	-0.233, 0.197	-0.203	-0.372, -0.033	-0.077	-0.247, 0.093	0.072	-0.163, 0.306	0.081	-0.165, 0.326	-0.005	-0.194, 0.184
Mid-PGY2	White	Female	0.048	-0.059, 0.154	-0.048	-0.138, 0.043	0.004	-0.082, 0.090	-0.005	-0.117, 0.107	0.103	-0.020, 0.227	0.018	-0.074, 0.110
Mid-PGY2	Asian	Male	-0.084	-0.227, 0.060	0.020	-0.102, 0.142	-0.058	-0.174, 0.058	-0.014	-0.166, 0.137	-0.040	-0.207, 0.127	-0.036	-0.160, 0.088
Mid-PGY2	Asian	Female	-0.140	-0.334, 0.054	-0.243	-0.407, -0.079	-0.071	-0.228, 0.085	-0.075	-0.279, 0.129	-0.032	-0.257, 0.193	-0.038	-0.205, 0.130
Mid-PGY2	URM	Male	0.001	-0.164, 0.166	-0.167	-0.308, -0.027	-0.130	-0.263, 0.004	-0.098	-0.272, 0.076	-0.036	-0.226, 0.155	-0.043	-0.185, 0.100
Mid-PGY2	URM	Female	-0.041	-0.232, 0.150	-0.271	-0.433, -0.109	-0.102	-0.257, 0.052	-0.039	-0.240, 0.163	0.025	-0.196, 0.245	-0.040	-0.205, 0.125

© 2023 Lett E et al. *JAMA Netw Open.*

End-PGY2	White	Female	0.044	-0.055, 0.143	-0.069	-0.175, 0.037	-0.006	-0.089, 0.077	-0.017	-0.128, 0.094	0.092	-0.024, 0.208	0.007	-0.078, 0.093
End-PGY2	Asian	Male	-0.113	-0.246, 0.020	-0.035	-0.178, 0.107	-0.071	-0.183, 0.041	-0.050	-0.199, 0.100	-0.067	-0.224, 0.089	-0.049	-0.165, 0.067
End-PGY2	Asian	Female	-0.152	-0.332, 0.028	-0.314	-0.507, -0.122	-0.102	-0.254, 0.050	-0.121	-0.324, 0.081	-0.077	-0.288, 0.135	-0.061	-0.218, 0.095
End-PGY2	URM	Male	-0.026	-0.179, 0.127	-0.266	-0.429, -0.103	-0.141	-0.270, -0.011	-0.161	-0.333, 0.011	-0.069	-0.248, 0.109	-0.063	-0.196, 0.070
End-PGY2	URM	Female	-0.064	-0.242, 0.113	-0.339	-0.528, -0.150	-0.128	-0.278, 0.021	-0.149	-0.348, 0.051	-0.032	-0.238, 0.175	-0.075	-0.229, 0.079
Mid-PGY3	White	Female	0.040	-0.057, 0.138	-0.090	-0.224, 0.044	-0.015	-0.102, 0.072	-0.030	-0.158, 0.098	0.080	-0.036, 0.196	-0.003	-0.092, 0.085
Mid-PGY3	Asian	Male	-0.142	-0.273, -0.010	-0.091	-0.271, 0.090	-0.084	-0.201, 0.034	-0.085	-0.257, 0.087	-0.094	-0.251, 0.063	-0.062	-0.181, 0.057
Mid-PGY3	Asian	Female	-0.165	-0.343, 0.014	-0.386	-0.630, -0.142	-0.133	-0.292, 0.026	-0.168	-0.401, 0.065	-0.122	-0.333, 0.090	-0.085	-0.246, 0.077
Mid-PGY3	URM	Male	-0.054	-0.205, 0.097	-0.364	-0.569, -0.158	-0.151	-0.286, -0.016	-0.224	-0.422, -0.027	-0.103	-0.281, 0.076	-0.083	-0.220, 0.054
Mid-PGY3	URM	Female	-0.088	-0.263, 0.088	-0.406	-0.645, -0.168	-0.154	-0.310, 0.002	-0.259	-0.488, -0.030	-0.088	-0.294, 0.119	-0.110	-0.269, 0.048
End-PGY3	White	Female	0.036	-0.067, 0.140	-0.112	-0.280, 0.057	-0.024	-0.121, 0.072	-0.042	-0.199, 0.115	0.069	-0.055, 0.192	-0.014	-0.113, 0.085
End-PGY3	Asian	Male	-0.171	-0.311, -0.031	-0.146	-0.374, 0.081	-0.097	-0.227, 0.034	-0.120	-0.332, 0.091	-0.121	-0.288, 0.046	-0.075	-0.209, 0.059
End-PGY3	Asian	Female	-0.177	-0.366, 0.012	-0.458	-0.765, -0.150	-0.163	-0.340, 0.014	-0.215	-0.501, 0.072	-0.166	-0.392, 0.059	-0.108	-0.289, 0.073
End-PGY3	URM	Male	-0.082	-0.242, 0.079	-0.462	-0.720, -0.204	-0.162	-0.312, -0.013	-0.288	-0.529, -0.047	-0.136	-0.327, 0.054	-0.103	-0.257, 0.050
End-PGY3	URM	Female	-0.111	-0.296, 0.075	-0.474	-0.774, -0.174	-0.180	-0.353, -0.007	-0.369	-0.649, -0.089	-0.144	-0.364, 0.076	-0.146	-0.324, 0.032

PGY: postgraduate year; CI: confidence interval

© 2023 Lett E et al. *JAMA Netw Open.*

eTable 8. Adjusted Differences in Mean Competency Scores Over the Evaluation Period for US Emergency Residents in *Four-Year Programs* by Each Ethnoracial Group and Sex Cross-Strata

Assessment Period	Racial Groups	Sex	Interpersonal and Communication Skills		Medical Knowledge		Patient Care		Practice-Based Learning and Improvement		Professionalism		System-Based Practice	
			β	95% CI	β	95% CI	β	95% CI	β	95% CI	β	95% CI	β	95% CI
Mid-PGY1	White	Female	0.006	-0.339, 0.350	-0.076	-0.258, 0.106	-0.037	-0.283, 0.208	-0.054	-0.230, 0.121	0.000	-0.305, 0.306	-0.011	-0.235, 0.212
Mid-PGY1	Asian	Male	-0.126	-0.537, 0.285	0.002	-0.211, 0.215	-0.124	-0.418, 0.169	0.051	-0.155, 0.257	-0.092	-0.456, 0.273	-0.023	-0.289, 0.242
Mid-PGY1	Asian	Female	0.046	-0.470, 0.561	-0.290	-0.554, -0.026	-0.028	-0.396, 0.341	0.003	-0.252, 0.259	-0.032	-0.489, 0.425	0.089	-0.244, 0.421
Mid-PGY1	URM	Male	-0.034	-0.503, 0.436	-0.179	-0.425, 0.068	-0.102	-0.437, 0.233	-0.054	-0.292, 0.183	-0.121	-0.538, 0.296	0.041	-0.263, 0.344
Mid-PGY1	URM	Female	0.092	-0.449, 0.633	-0.424	-0.706, -0.141	0.014	-0.373, 0.400	-0.075	-0.348, 0.197	0.065	-0.416, 0.545	-0.062	-0.412, 0.288
End-PGY1	White	Female	0.008	-0.287, 0.304	-0.093	-0.254, 0.069	-0.037	-0.249, 0.175	-0.049	-0.201, 0.102	0.009	-0.256, 0.273	-0.006	-0.199, 0.186
End-PGY1	Asian	Male	-0.124	-0.477, 0.228	-0.004	-0.194, 0.187	-0.111	-0.364, 0.143	0.055	-0.123, 0.234	-0.087	-0.403, 0.229	-0.018	-0.247, 0.211
End-PGY1	Asian	Female	0.011	-0.432, 0.454	-0.269	-0.505, -0.033	-0.049	-0.368, 0.269	-0.014	-0.236, 0.208	-0.035	-0.431, 0.361	0.045	-0.243, 0.332
End-PGY1	URM	Male	-0.054	-0.458, 0.350	-0.179	-0.399, 0.041	-0.105	-0.395, 0.185	-0.067	-0.274, 0.139	-0.144	-0.505, 0.218	0.018	-0.244, 0.281
End-PGY1	URM	Female	0.034	-0.431, 0.498	-0.449	-0.701, -0.198	-0.033	-0.367, 0.301	-0.122	-0.358, 0.114	-0.017	-0.434, 0.399	-0.092	-0.394, 0.211
Mid-PGY2	White	Female	0.011	-0.239, 0.262	-0.110	-0.266, 0.046	-0.036	-0.218, 0.145	-0.044	-0.183, 0.094	0.017	-0.211, 0.245	-0.001	-0.166, 0.163
Mid-PGY2	Asian	Male	-0.123	-0.422, 0.176	-0.009	-0.196, 0.177	-0.097	-0.315, 0.120	0.059	-0.106, 0.224	-0.082	-0.355, 0.191	-0.013	-0.210, 0.184
Mid-PGY2	Asian	Female	-0.023	-0.398, 0.353	-0.247	-0.480, -0.015	-0.071	-0.344, 0.202	-0.031	-0.237, 0.174	-0.038	-0.381, 0.305	0.000	-0.246, 0.247
Mid-PGY2	URM	Male	-0.074	-0.417, 0.269	-0.179	-0.394, 0.036	-0.108	-0.356, 0.141	-0.080	-0.271, 0.110	-0.167	-0.479, 0.146	-0.004	-0.230, 0.222
Mid-PGY2	URM	Female	-0.024	-0.418, 0.369	-0.475	-0.720, -0.230	-0.079	-0.365, 0.207	-0.168	-0.385, 0.049	-0.099	-0.459, 0.260	-0.121	-0.381, 0.138

© 2023 Lett E et al. *JAMA Netw Open.*

End-PGY2	White	Female	0.014	-0.196, 0.225	-0.127	-0.295, 0.041	-0.036	-0.191, 0.119	-0.039	-0.179, 0.101	0.026	-0.173, 0.224	0.004	-0.138, 0.146
End-PGY2	Asian	Male	-0.121	-0.374, 0.131	-0.015	-0.218, 0.188	-0.084	-0.270, 0.103	0.063	-0.105, 0.232	-0.077	-0.316, 0.162	-0.007	-0.178, 0.163
End-PGY2	Asian	Female	-0.057	-0.374, 0.260	-0.226	-0.480, 0.027	-0.093	-0.327, 0.141	-0.049	-0.259, 0.162	-0.041	-0.341, 0.259	-0.044	-0.257, 0.170
End-PGY2	URM	Male	-0.095	-0.385, 0.196	-0.179	-0.411, 0.053	-0.110	-0.324, 0.103	-0.093	-0.287, 0.100	-0.189	-0.463, 0.085	-0.027	-0.222, 0.169
End-PGY2	URM	Female	-0.083	-0.414, 0.249	-0.500	-0.765, -0.236	-0.126	-0.370, 0.119	-0.214	-0.435, 0.006	-0.181	-0.495, 0.133	-0.151	-0.375, 0.073
Mid-PGY3	White	Female	0.017	-0.164, 0.198	-0.144	-0.338, 0.050	-0.035	-0.171, 0.100	-0.034	-0.190, 0.121	0.034	-0.146, 0.214	0.009	-0.118, 0.136
Mid-PGY3	Asian	Male	-0.120	-0.338, 0.098	-0.021	-0.255, 0.214	-0.070	-0.234, 0.094	0.067	-0.121, 0.256	-0.072	-0.290, 0.146	-0.002	-0.156, 0.151
Mid-PGY3	Asian	Female	-0.091	-0.364, 0.182	-0.205	-0.499, 0.089	-0.115	-0.320, 0.091	-0.066	-0.302, 0.170	-0.044	-0.317, 0.229	-0.088	-0.280, 0.105
Mid-PGY3	URM	Male	-0.115	-0.365, 0.136	-0.179	-0.447, 0.088	-0.113	-0.301, 0.074	-0.107	-0.322, 0.108	-0.212	-0.461, 0.037	-0.049	-0.225, 0.127
Mid-PGY3	URM	Female	-0.141	-0.426, 0.145	-0.526	-0.831, -0.221	-0.172	-0.387, 0.043	-0.261	-0.506, -0.015	-0.263	-0.548, 0.022	-0.181	-0.382, 0.020
End-PGY3	White	Female	0.020	-0.146, 0.186	-0.161	-0.391, 0.068	-0.035	-0.161, 0.092	-0.029	-0.211, 0.153	0.042	-0.133, 0.218	0.014	-0.108, 0.136
End-PGY3	Asian	Male	-0.118	-0.319, 0.083	-0.027	-0.304, 0.250	-0.056	-0.210, 0.097	0.071	-0.148, 0.291	-0.067	-0.280, 0.146	0.003	-0.145, 0.151
End-PGY3	Asian	Female	-0.125	-0.377, 0.126	-0.184	-0.532, 0.164	-0.136	-0.329, 0.056	-0.083	-0.359, 0.193	-0.047	-0.314, 0.220	-0.132	-0.317, 0.054
End-PGY3	URM	Male	-0.135	-0.366, 0.096	-0.180	-0.494, 0.135	-0.116	-0.292, 0.059	-0.120	-0.370, 0.131	-0.235	-0.478, 0.009	-0.072	-0.241, 0.098
End-PGY3	URM	Female	-0.199	-0.461, 0.064	-0.552	-0.912, -0.192	-0.218	-0.419, -0.018	-0.307	-0.593, -0.021	-0.345	-0.623, -0.067	-0.211	-0.404, -0.017
Mid-PGY4	White	Female	0.023	-0.147, 0.194	-0.178	-0.448, 0.092	-0.034	-0.164, 0.095	-0.024	-0.239, 0.190	0.051	-0.135, 0.237	0.019	-0.110, 0.148
Mid-PGY4	Asian	Male	-0.117	-0.323, 0.090	-0.032	-0.358, 0.293	-0.043	-0.200, 0.115	0.075	-0.183, 0.334	-0.062	-0.288, 0.164	0.008	-0.148, 0.165
Mid-PGY4	Asian	Female	-0.159	-0.418, 0.099	-0.162	-0.572, 0.247	-0.158	-0.355, 0.039	-0.100	-0.426, 0.225	-0.050	-0.333, 0.233	-0.176	-0.372, 0.020
Mid-PGY4	URM	Male	-0.155	-0.392, 0.082	-0.180	-0.549, 0.190	-0.119	-0.299, 0.060	-0.133	-0.426, 0.161	-0.257	-0.515, 0.000	-0.094	-0.273, 0.085

© 2023 Lett E et al. *JAMA Netw Open.*

Mid-PGY4	URM	Female	-0.257	-0.526, 0.012	-0.577	-1.001, -0.154	-0.265	-0.470, -0.060	-0.353	-0.690, -0.017	-0.427	-0.721, -0.132	-0.240	-0.445, -0.036
End-PGY4	White	Female	0.026	-0.167, 0.219	-0.195	-0.510, 0.119	-0.034	-0.178, 0.111	-0.019	-0.270, 0.232	0.059	-0.151, 0.269	0.024	-0.122, 0.170
End-PGY4	Asian	Male	-0.115	-0.348, 0.117	-0.038	-0.417, 0.340	-0.029	-0.204, 0.146	0.080	-0.223, 0.382	-0.057	-0.311, 0.197	0.014	-0.163, 0.190
End-PGY4	Asian	Female	-0.194	-0.485, 0.098	-0.141	-0.617, 0.334	-0.180	-0.399, 0.039	-0.118	-0.498, 0.263	-0.053	-0.371, 0.265	-0.220	-0.441, 0.001
End-PGY4	URM	Male	-0.176	-0.442, 0.091	-0.180	-0.609, 0.249	-0.122	-0.322, 0.077	-0.146	-0.489, 0.198	-0.280	-0.569, 0.009	-0.117	-0.317, 0.084
End-PGY4	URM	Female	-0.315	-0.618, -0.012	-0.603	-1.095, -0.111	-0.311	-0.539, -0.083	-0.400	-0.793, -0.006	-0.509	-0.840, -0.178	-0.270	-0.500, -0.040

PGY: postgraduate year; CI: confidence interval

© 2023 Lett E et al. *JAMA Netw Open.*

eTable 9. Mean Evaluation Scores for Three-Year and Four-Year Programs Over the Study Period by Each Ethnoracial Group and Sex Cross-Strata

Assessment Period	Racial Groups	Sex	Three-Year Programs	Four-Year Programs
			Mean (SD)	Mean (SD)
Mid-PGY1	White	Male	2.37 (0.72)	2.01 (0.72)
Mid-PGY1	White	Female	2.45 (0.73)	2.01 (0.70)
Mid-PGY1	Asian	Male	2.35 (0.79)	2.02 (0.80)
Mid-PGY1	Asian	Female	2.43 (0.77)	2.03 (0.81)
Mid-PGY1	URM	Male	2.27 (0.86)	1.97 (0.75)
Mid-PGY1	URM	Female	2.44 (0.79)	2.29 (0.90)
End-PGY1	White	Male	3.36 (0.74)	2.91 (0.58)
End-PGY1	White	Female	3.45 (0.76)	2.86 (0.66)
End-PGY1	Asian	Male	3.33 (0.81)	2.90 (0.51)
End-PGY1	Asian	Female	3.29 (0.69)	2.79 (0.67)
End-PGY1	URM	Male	3.28 (0.88)	2.64 (0.55)
End-PGY1	URM	Female	3.35 (0.80)	2.95 (0.60)
Mid-PGY2	White	Male	4.42 (0.73)	3.79 (0.69)
Mid-PGY2	White	Female	4.46 (0.69)	3.75 (0.66)
Mid-PGY2	Asian	Male	4.30 (0.83)	3.77 (0.69)
Mid-PGY2	Asian	Female	4.24 (0.71)	3.66 (0.83)
Mid-PGY2	URM	Male	4.24 (0.74)	3.53 (0.86)
Mid-PGY2	URM	Female	4.20 (0.73)	3.61 (0.68)
End-PGY2	White	Male	5.20 (0.68)	4.61 (0.67)
End-PGY2	White	Female	5.21 (0.69)	4.50 (0.77)
End-PGY2	Asian	Male	5.05 (0.75)	4.52 (0.68)
End-PGY2	Asian	Female	4.98 (0.75)	4.55 (0.82)
End-PGY2	URM	Male	4.99 (0.76)	4.38 (0.84)
End-PGY2	URM	Female	5.00 (0.69)	4.33 (0.72)
Mid-PGY3	White	Male	6.06 (0.73)	5.38 (0.65)
Mid-PGY3	White	Female	6.11 (0.65)	5.34 (0.70)
Mid-PGY3	Asian	Male	5.95 (0.78)	5.38 (0.71)
Mid-PGY3	Asian	Female	5.89 (0.78)	5.31 (0.76)
Mid-PGY3	URM	Male	5.82 (0.77)	5.30 (0.83)
Mid-PGY3	URM	Female	5.94 (0.65)	5.19 (0.69)
End-PGY3	White	Male	6.84 (0.62)	5.99 (0.65)
End-PGY3	White	Female	6.88 (0.54)	5.95 (0.65)
End-PGY3	Asian	Male	6.72 (0.60)	5.93 (0.66)
End-PGY3	Asian	Female	6.71 (0.59)	5.97 (0.57)
End-PGY3	URM	Male	6.56 (0.77)	5.80 (0.68)
End-PGY3	URM	Female	6.64 (0.70)	5.63 (0.68)
Mid-PGY4	White	Male	–	6.70 (0.67)
Mid-PGY4	White	Female	–	6.66 (0.59)
Mid-PGY4	Asian	Male	–	6.65 (0.55)

© 2023 Lett E et al. *JAMA Netw Open.*

Mid-PGY4	Asian	Female	–	6.57 (0.58)
Mid-PGY4	URM	Male	–	6.53 (0.67)
Mid-PGY4	URM	Female	–	6.33 (1.07)
End-PGY4	White	Male	–	7.20 (0.47)
End-PGY4	White	Female	–	7.17 (0.45)
End-PGY4	Asian	Male	–	7.16 (0.48)
End-PGY4	Asian	Female	–	7.08 (0.45)
End-PGY4	URM	Male	–	6.93 (0.53)
End-PGY4	URM	Female	–	6.92 (0.54)

PGY: postgraduate year; CI: confidence interval

© 2023 Lett E et al. *JAMA Netw Open.*

eTable 10. Adjusted Differences in Mean Evaluation Scores for Three-Year and Four-Year Programs Over the Study Period by Each Ethnoracial Group and Sex Cross-Strata

Assessment Period	Racial Groups	Sex	Three-Year Programs		Four-Year Programs	
			β	95% CI	β	95% CI
Mid-PGY1	White	Female	0.035	-0.068, 0.139	-0.020	-0.230, 0.190
Mid-PGY1	Asian	Male	0.035	-0.104, 0.175	-0.061	-0.311, 0.190
Mid-PGY1	Asian	Female	-0.025	-0.213, 0.163	-0.031	-0.345, 0.283
Mid-PGY1	URM	Male	0.013	-0.147, 0.172	-0.074	-0.360, 0.213
Mid-PGY1	URM	Female	0.013	-0.172, 0.198	-0.062	-0.393, 0.268
End-PGY1	White	Female	0.023	-0.068, 0.114	-0.019	-0.201, 0.164
End-PGY1	Asian	Male	0.006	-0.117, 0.129	-0.056	-0.274, 0.162
End-PGY1	Asian	Female	-0.064	-0.229, 0.102	-0.048	-0.321, 0.225
End-PGY1	URM	Male	-0.031	-0.172, 0.110	-0.086	-0.336, 0.163
End-PGY1	URM	Female	-0.040	-0.203, 0.123	-0.110	-0.397, 0.178
Mid-PGY2	White	Female	0.011	-0.072, 0.095	-0.018	-0.175, 0.140
Mid-PGY2	Asian	Male	-0.024	-0.137, 0.090	-0.052	-0.241, 0.138
Mid-PGY2	Asian	Female	-0.103	-0.255, 0.050	-0.066	-0.303, 0.172
Mid-PGY2	URM	Male	-0.075	-0.205, 0.055	-0.099	-0.316, 0.118
Mid-PGY2	URM	Female	-0.093	-0.244, 0.058	-0.157	-0.406, 0.092
End-PGY2	White	Female	-0.001	-0.084, 0.083	-0.017	-0.156, 0.122
End-PGY2	Asian	Male	-0.053	-0.166, 0.060	-0.047	-0.215, 0.120
End-PGY2	Asian	Female	-0.142	-0.294, 0.011	-0.083	-0.293, 0.127
End-PGY2	URM	Male	-0.118	-0.248, 0.011	-0.112	-0.304, 0.080
End-PGY2	URM	Female	-0.146	-0.296, 0.004	-0.204	-0.424, 0.016
Mid-PGY3	White	Female	-0.012	-0.102, 0.077	-0.016	-0.143, 0.112
Mid-PGY3	Asian	Male	-0.083	-0.204, 0.039	-0.043	-0.198, 0.112
Mid-PGY3	Asian	Female	-0.181	-0.345, -0.016	-0.101	-0.295, 0.093
Mid-PGY3	URM	Male	-0.162	-0.301, -0.023	-0.124	-0.302, 0.053
Mid-PGY3	URM	Female	-0.199	-0.360, -0.038	-0.252	-0.455, -0.049
End-PGY3	White	Female	-0.024	-0.126, 0.077	-0.015	-0.141, 0.112
End-PGY3	Asian	Male	-0.112	-0.249, 0.025	-0.038	-0.193, 0.116
End-PGY3	Asian	Female	-0.219	-0.405, -0.034	-0.118	-0.311, 0.075
End-PGY3	URM	Male	-0.206	-0.363, -0.049	-0.137	-0.313, 0.039
End-PGY3	URM	Female	-0.252	-0.434, -0.070	-0.299	-0.500, -0.098
Mid-PGY4	White	Female	–	–	-0.014	-0.150, 0.122
Mid-PGY4	Asian	Male	–	–	-0.034	-0.199, 0.131
Mid-PGY4	Asian	Female	–	–	-0.136	-0.343, 0.071
Mid-PGY4	URM	Male	–	–	-0.149	-0.337, 0.039
Mid-PGY4	URM	Female	–	–	-0.347	-0.561, -0.132
End-PGY4	White	Female	–	–	-0.013	-0.166, 0.141
End-PGY4	Asian	Male	–	–	-0.029	-0.215, 0.156
End-PGY4	Asian	Female	–	–	-0.153	-0.386, 0.079
End-PGY4	URM	Male	–	–	-0.162	-0.373, 0.049
End-PGY4	URM	Female	–	–	-0.394	-0.636, -0.152

PGY: postgraduate year; CI: confidence interval

© 2023 Lett E et al. *JAMA Netw Open.*

eFigure 1. Analytic Cohort Development and Exclusion Criteria

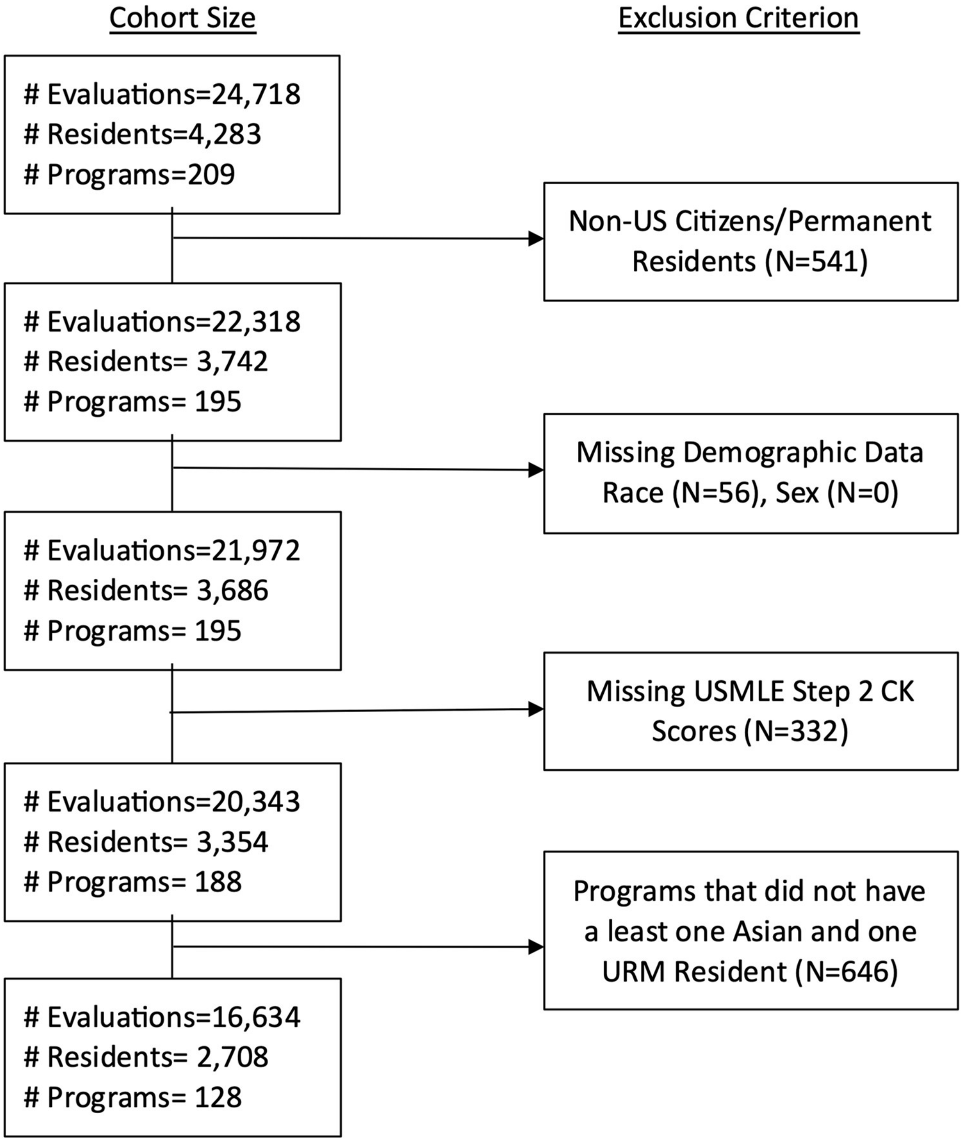

*N indicates the number of residents whose data were excluded based on the corresponding criterion.

© 2023 Lett E et al. *JAMA Netw Open.*

eFigure 2. Mean Competency Scores for the Overall Sample. Changes in the mean scores for 6 different ACGME competencies among US emergency medicine residents in three-year programs and four-year programs. Mean competency scores are calculated as the mean of each subcompetency scores corresponding to each competency.

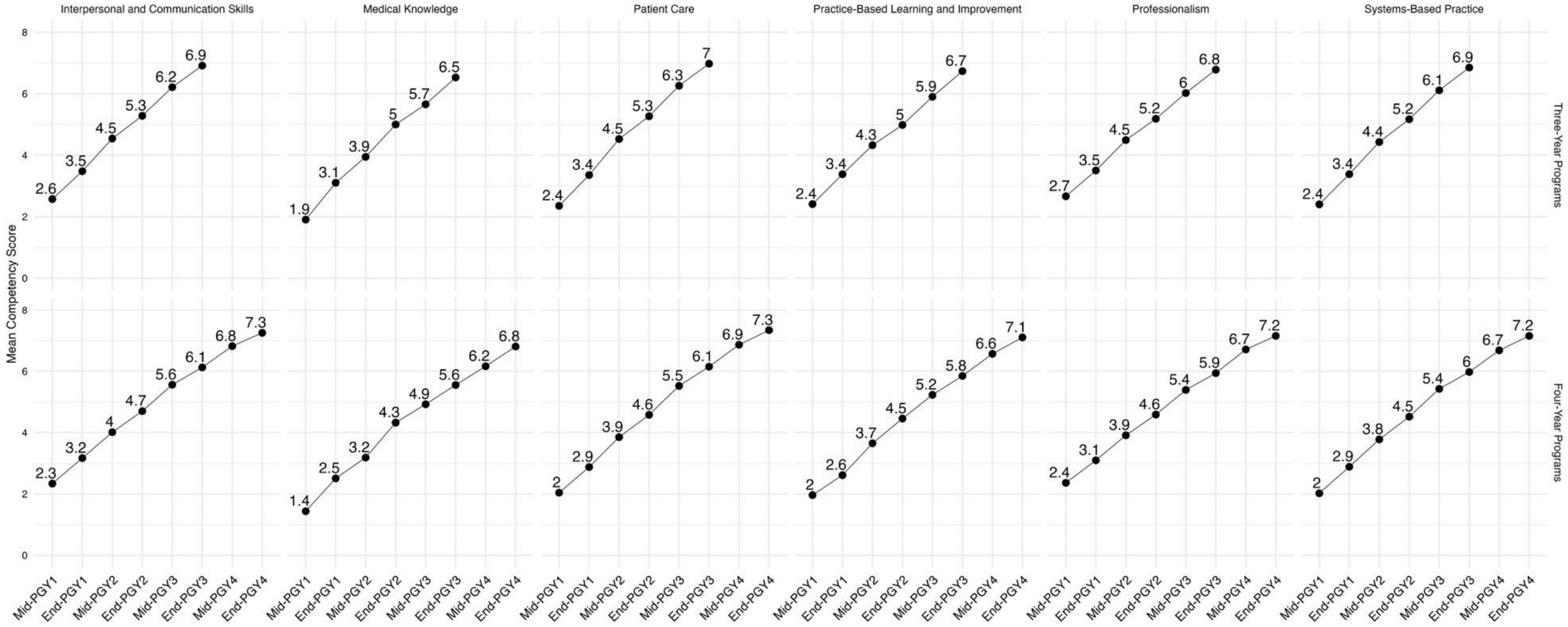

© 2023 Lett E et al. *JAMA Netw Open.*

eFigure 3. Mean Assessment Score for the Overall Sample. Changes in the mean assessment score among emergency medicine residents in the United States by program length. Mean assessment scores are calculated as the mean of the mean 6 competencies scores for the entire sample.

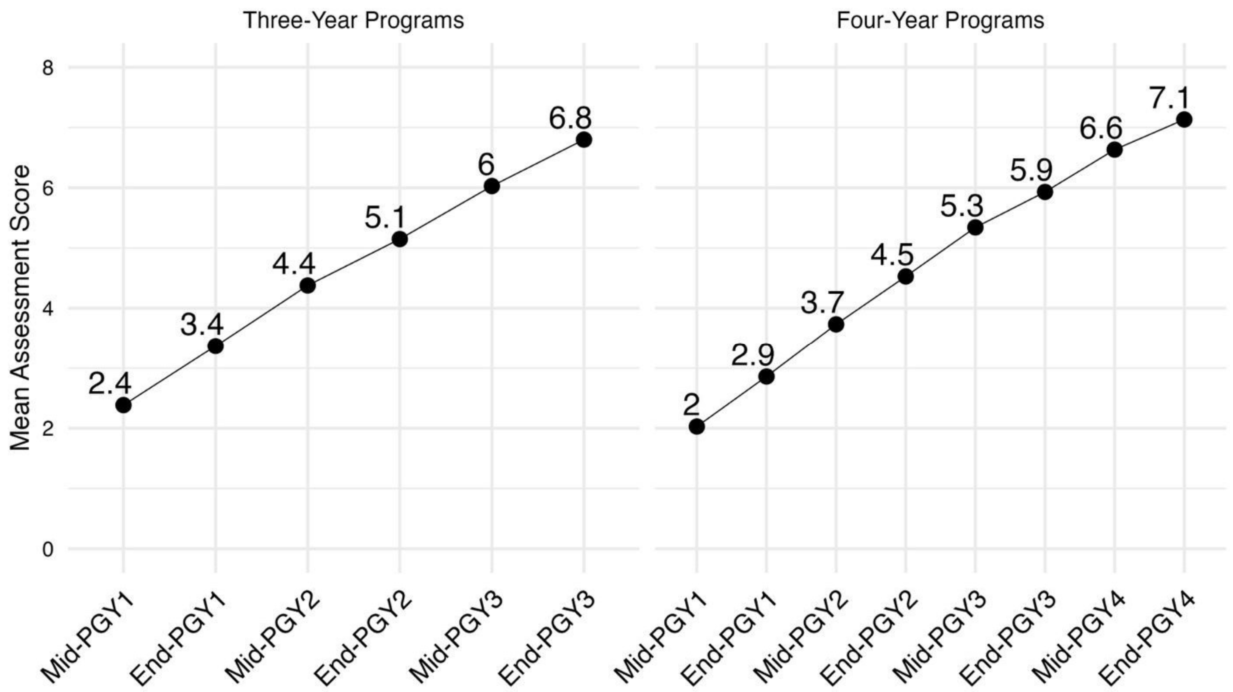

© 2023 Lett E et al. *JAMA Netw Open.*

eFigure 4. Assessment Score Trajectories by Ethnoracial Group and Sex Cross-Strata. Changes in the mean assessment score among US emergency medicine residents in three-year and four-year programs. Mean assessment scores are calculated as the mean of the mean 6 competencies scores for each Ethnoracial Group and sex cross-strata stratified by length of program. URM: underrepresented in medicine.

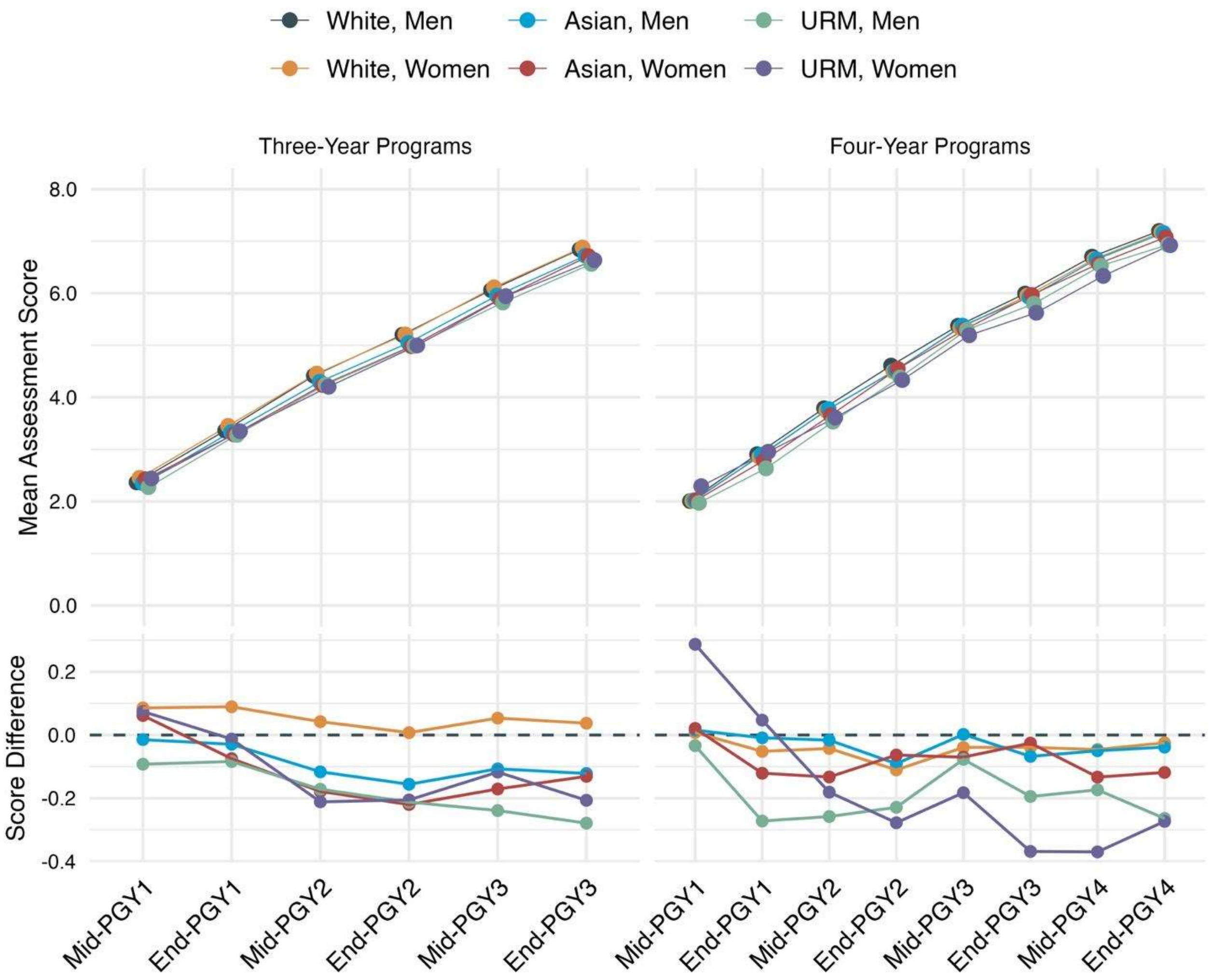

© 2023 Lett E et al. *JAMA Netw Open.*
