# Supplementary material for: Intersectional Disparities in Emergency Medicine Residents’ Performance Assessments by Race, Ethnicity, and Sex
Source: JAMA Netw Open. 2023 Sep 21;6(9):e2330847. doi: 10.1001/jamanetworkopen.2023.30847 (PMC10514741; doi:10.1001/jamanetworkopen.2023.30847)
Supplement: Supplement 2. — Data Sharing Statement [file jamanetwopen-e2330847-s002.pdf]

Data Sharing Statement

Lett. Intersectional Disparities in Emergency Medicine Residents' Performance Assessments by Race, Ethnicity, and Sex. *JAMA Netw Open*. Published September 11, 2023. doi:10.1001/jamanetworkopen.2023.30847

Data
Data available: No

Additional Information
Explanation for why data not available: This data is sensitive data provided by the Association of American Medical Colleges and Accreditation Council of Graduate Medical Education and cannot be made public for trainee privacy and safety.
